# *Picomonas judraskeda* Gen. Et Sp. Nov.: The First Identified Member of the Picozoa Phylum Nov., a Widespread Group of Picoeukaryotes, Formerly Known as ‘Picobiliphytes’

**DOI:** 10.1371/journal.pone.0059565

**Published:** 2013-03-26

**Authors:** Ramkumar Seenivasan, Nicole Sausen, Linda K. Medlin, Michael Melkonian

**Affiliations:** 1 Department of Botany, Cologne Biocenter, University of Cologne, Cologne, Germany; 2 Marine Biological Association of the UK, The Laboratory, The Citadel, Plymouth, United Kingdom; University of Melbourne, Australia

## Abstract

In 2007, a novel, putatively photosynthetic picoeukaryotic lineage, the ‘picobiliphytes’, with no known close eukaryotic relatives, was reported from 18S environmental clone library sequences and fluorescence in situ hybridization. Although single cell genomics later showed these organisms to be heterotrophic rather than photosynthetic, until now this apparently widespread group of pico-(or nano-)eukaryotes has remained uncultured and the organisms could not be formally recognized. Here, we describe *Picomonas judraskeda* gen. et sp. nov., from marine coastal surface waters, which has a ‘picobiliphyte’ 18S rDNA signature. Using vital mitochondrial staining and cell sorting by flow cytometry, a single cell-derived culture was established. The cells are biflagellate, 2.5–3.8×2–2.5 µm in size, lack plastids and display a novel stereotypic cycle of cell motility (described as the “jump, drag, and skedaddle”-cycle). They consist of two hemispherical parts separated by a deep cleft, an anterior part that contains all major cell organelles including the flagellar apparatus, and a posterior part housing vacuoles/vesicles and the feeding apparatus, both parts separated by a large vacuolar cisterna. From serial section analyses of cells, fixed at putative stages of the feeding cycle, it is concluded that cells are not bacterivorous, but feed on small marine colloids of less than 150 nm diameter by fluid-phase, bulk flow endocytosis. Based on the novel features of cell motility, ultrastructure and feeding, and their isolated phylogenetic position, we establish a new phylum, Picozoa, for *Picomonas judraskeda*, representing an apparently widespread and ecologically important group of heterotrophic picoeukaryotes, formerly known as ‘picobiliphytes’.

## Introduction

Microbial plankton plays a pivotal role in global biogeochemical processes and provides amenities and services that are essential to mankind’s existence, including food production, remediation of waste and regulation of aspects of the climate in the biosphere [1]–[4]. Picoplankton, organisms that can pass through filters of 2 µm [5] or 3 µm [6], are widespread and dominate the biomass in the aquatic environment [7]–[9]. Although initially focusing on phototrophic picoprokaryotes, the use of molecular techniques to directly analyze gene sequences in natural samples was also applied to very small eukaryotes in three seminal studies in 2001 that revealed an unexpectedly large and novel diversity of picoeukaryotes [10]–[12]. Picoeukaryotes are now known to be ubiquitous in surface waters of all oceans and are likely the most abundant eukaryotes in the sea [6], [13]–[15]. When defined as cells <3 µm, picoeukaryotes consist of the numerically more abundant phototrophic and the less abundant heterotrophic picoeukaryotes (especially in oligotrophic coastal sites; [16]). The latter are generally regarded as bacterial grazers [13] although some are herbivores feeding on picoplanktonic cyanobacteria or photosynthetic picoeukaryotes [17], and others are parasites [18].

A large fraction of the *in-situ* diversity of picoeukaryotes has not been studied in culture and these organisms are often regarded as ‘unculturable’ [15]. Thus, various methods to address the function of uncultured picoeukaryotes have been developed in recent years. One approach has been the application of specific oligonucleotide probes for fluorescence *in-situ* hybridization (FISH) often coupled with flow cytometry [19]–[22]. Furthermore, direct sequencing of community DNA or RNA (metagenomics, metatranscriptomics) reveals gene repertoires and metabolic functions [23], and targeted metagenomics has recently been applied to uncultured picoeukaryotes [24], [25]. Another approach has been to stimulate the growth of heterotrophic picoeukaryotes by incubation in unamended seawater in the dark resulting in the identification of previously unknown organisms [26]–[28]. Finally, sorting of single picoeukaryotic cells [29], [30] has recently opened possibilities for singe cell genomics (SCG) by whole genome amplification and next-generation sequencing of sorted cells thus coupling molecular identification to metabolic functions [31]–[33].

Most of these methods have also been applied to a widely distributed group of uncultured picoeukaryotes that represent a deep evolutionary branch without clear affinities to other eukaryotes. Initially described from cold and polar waters using 18S rDNA clone libraries and FISH, as a group of picoeukaryotic phycobilin-containing algae with affinities to cryptophytes and katablepharids and named ‘picobiliphytes’ [34], [35], these organisms were subsequently also found in subtropical and tropical surface waters using the same methods [36]. The latter authors, however, reported that ‘picobiliphytes’ were of nanoplanktonic rather than picoplanktonic size, and thus renamed them ‘biliphytes’; they also concluded that in tropical eddy-influenced surface waters ‘biliphytes’ contributed about 30% of the phytoplanktonic biomass. ‘(Pico)biliphyte’ sequences were also detected in surface waters of other oceans, such as the South-East Pacific Ocean [37], the South China Sea [38], the Indian Ocean [39], and the brackish Baltic Sea [28]. Another study using the same FISH probes originally applied to identify ‘(pico)biliphytes’ on filtered samples from the North Pacific failed to co-localize phycoerythrin with the hybridized cells and concluded that these picoeukaryotes were likely not obligate photoautotrophs but rather mixotrophs or phagotrophs and the presence of orange (phycoerythrin) fluorescence in such cells could represent ingested prey (e.g. picocyanobacteria, [40]). This uncertainty of whether ‘(pico)biliphytes’ were autotrophic or heterotrophic was dispelled by a study that used single cell genomics on three ‘(pico)biliphyte’ cells (from the three clades identified by Not et al. 2007, [34]) isolated by FACS (fluorescence-activated cell sorting) with a lysosomal marker [41]. The authors did not find evidence of plastid DNA or of nuclear-encoded plastid-targeted proteins in the partially sequenced genomes of the three cells and concluded that ‘(pico)biliphytes’ were likely heterotrophic. Furthermore, they found both viral and bacterial DNA associated with the cells, which led them to conclude that ‘(pico)biliphytes’ may feed on bacteria and large DNA viruses, although viral infections as well as passive attachment of bacteria and viruses to the cells could not be excluded. Although all these studies provided important information about this ‘uncultured’ group of heterotrophic pico- or nanoeukaryotes, the cells themselves have never been seen alive nor studied in detail by light and electron microscopy.

Here we used a fluorescent mitochondrial marker to isolate single ‘(pico)biliphyte’ cells by FACS and established a first culture of these organisms. A detailed light and electron microscopic study based on this culture allowed us to describe a new genus and species (*Picomonas judraskeda* gen. nov., sp. nov.), and to formally erect a new phylum (Picozoa phylum nov.) for these enigmatic picoeukaryotes. The cells displayed many behavioral and structural features that, to our knowledge, have not yet been observed in any other eukaryotes and presumably relate to the cells’ specific feeding strategy. We verify that cells are heterotrophic and lack plastids, and conclude that the species studied feeds on small-size marine colloids (<150 nm) using a feeding strategy that we describe as a fluid-phase, bulk flow mechanism.

## Materials and Methods

### Culturing and Fluorescence-activated Cell Sorting

About 600 ml of surface seawater (5 m depth) was collected from the North Sea (Helgoland Roads, 54°11′N, 7°54′E, Germany) in August 2008 and successively filtered through 10 µm and 2 µm Isopore membrane filters (Millipore). From the filtrate, 50 ml was centrifuged at 4000 *g* in Falcon centrifuge tubes for 10 min at 15°C (Multifuge 3SR+, Heraeus). Cells from the supernatant were collected on a 0.2 µm membrane filter and directly transferred into a culture flask containing 0.1 µm **F**ilter- **S**terilized **S**ea **W**ater (FSSW), the inconspicuous pellet was resuspended with FSSW. Both samples were incubated at 15°C in a 14/10 hour L/D cycle with 9 µmol*m^−2^*s^−1^ photon fluence rate. Cells were inoculated at regular intervals (every two weeks) into fresh FSSW. Presence of ‘(pico)biliphytes’ was tested by amplification of nuclear-encoded small subunit rDNA (18S rDNA) by Polymerase Chain Reaction (PCR) with ‘(pico)biliphyte’-specific primers (PICOBI01F and P01ITS1R, Table S1). In addition, the universal 18S eukaryote-specific reverse rDNA primer (1055R, Table S1) was used along with PICOBI01F for secondary amplification if required, and amplicons were confirmed by DNA sequencing. Cycle sequencing was performed with BigDye® Terminator V1.3 (Applied Biosystems) at the Cologne Centre for Genomics (University of Cologne).

**Figure 1:**
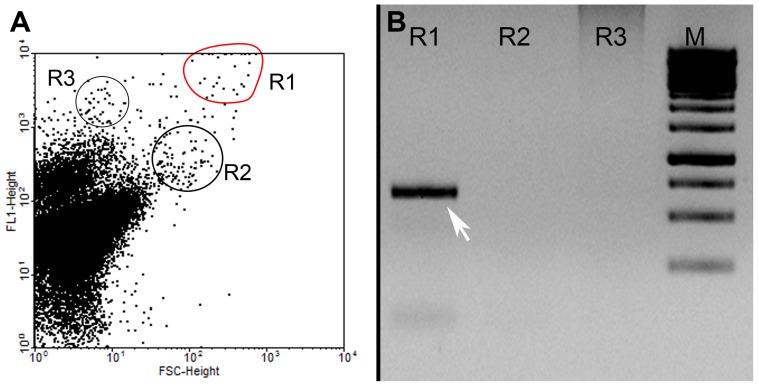
Identification and isolation of ‘(pico)biliphytes’ by fluorescence-activated cell sorting (*MitoTracker®* Green FM [FL1] vs. Forward Scatter [FSC]) and PCR amplification using specific primers. 1A. Approximately 100 cells from each of three regions with higher fluorescence/forward scatter (R1–R3) were sorted into PCR tubes. Cells marked in ‘red’ in the cytogram corresponded to ‘(pico)biliphytes’ by PCR amplification and sequencing. 1B. Sorted cells used for primary amplification with ‘picobiliphyte’-specific primers (PICOBI01F/P01ITS1R) and reamplified with PICOBI01F and 1055rev. Amplicons in region R1 were confirmed by DNA sequencing to correspond to ‘(pico)biliphytes’. Arrow 650 base pairs; M 1 Kb ladder.

A ‘(pico)biliphyte’-positive enrichment culture was prepared for fluorescence-activated cell sorting by adding 20 nM MitoTracker® Green FM (M7514, Invitrogen) to 10 ml of sample and incubated at 15°C in the dark for 15–20 min. Cells were sorted with a FACSvantage^SE^ (Becton Dickinson, NJ) using an Argon laser at 488 nm. Cells with high green fluorescence and forward scatter were targeted and sorted directly into PCR tubes for further confirmation of ‘(pico)biliphytes’ by PCR amplification and DNA sequencing. Single cell sorting was performed for the targeted region (Fig. 1; positive to ‘(pico)biliphytes’) into 24-well plates and plates were incubated at 15°C as described above. Single cell-derived cultures were rechecked for the presence of ‘(pico)biliphytes’ by PCR with universal eukaryotic rDNA primers (1F, BR, Table S1) and confirmed by sequencing. Cells were regularly (usually every 10–14 days) transferred into 10 ml of fresh FSSW in tissue culture flasks (25 or 50 ml) with 20 µl of soil extract [42] and monitored by light microscopy using phase contrast optics.

### Fluorescence Microscopy

For fluorescence microscopy (FL), cells (from a 10 day old culture) were fixed with 1.25% (v/v, final concentration) glutaraldehyde (GA) in culture medium for 30 min on ice. Fixed cells were directly placed on a glass slide pre-coated with poly L-lysine (P8920, Sigma) and allowed to settle for 30 min at room temperature. For FL, cells were probed with *MitoTracker®* Red CMXRos (Invitrogen, Darmstadt, Germany) for 10 min. Cells were washed with saline ethanol (22 ml of 100% ethanol, 5 ml of deionized water, 3 ml of 25X SET [3.75 M NaCl, 25 mM EDTA, 0.5 M Tris–HCl and pH 7.8]). DAPI (4′, 6-diamidino-2-phenylindole; 2 µg/ml final conc.) was used for nuclear staining.

### Electron Microscopy

For transmission electron microscopy, a cell suspension (10 ml) was fixed with a mixture of 0.32% freshly prepared para-formaldehyde (PFA), 0.125% glutaraldehyde (GA) and 0.01% of osmium tetroxide (v/v, final concentration), and incubated on ice for 30 min. The fixed cells were subsequently transferred to 1.5 ml centrifuge tubes (pre-coated with dichlorodimethylsilane (40410, Fluka, Buchs, Switzerland)) and pelleted at 4000 *g* for 10 min. at 15°C (this step was repeated until a visible pellet was seen). The pellet was washed and centrifuged twice with 500 µl of FSSW. Bovine serum albumin (50% BSA, A6063-Sigma) was added to coat the pellet. The supernatant was removed and 50 µl of 1.25% GA (v/v) in FSSW was overlaid to fix the pellet with BSA, and incubated on ice for 30 min. The translucent pellet was carefully transferred to a new 1.5 ml centrifuge tube for dehydration. Before dehydration, the salinity of the pellet was slowly reduced by 50% and 25% of FSSW with 12.5% and 22.5% of ethanol in water (v/v final conc.) respectively. Dehydration, embedding and serial sectioning were performed as described in Geimer and Melkonian, 2004 [43]. A total of 52 cells were serially sectioned (minimum number of 20 sections per cell of 60 nm thickness each), of which 8 cells were completely sectioned. Electron micrographs were taken with a Philips CM10 transmission electron microscope using a Gatan ORIUS TEM CCD camera and Digital Micrograph software. Further analysis of electron micrographs was carried out in Photoshop (Adobe CS4, version 11.0.2). For 3 D-modeling of *P. judraskeda* cells, images from serial sections were initially aligned with Adobe Photoshop (CS4), and later imported into IMOD 4.1.10 [44] software. The final 3 D -graphical animation of *P. judraskeda* cell (Movie S2, Movie S3) was created by Blender v.2.63 modeling software (http://www.blender.org/).

For scanning electron microscopy; cells were fixed with 1% PFA and 1.25% GA (v/v) for 30 min in culture medium. Fixed cells were directly placed on a glass slide, coated with poly L-lysine and allowed to settle for 30 min. Cells were gradually washed to decrease the salinity with 100%, 50% and 25% (v/v) FSSW followed by dehydration as in Martin-Cereceda et al. (2009) except that the last critical point drying step was with liquid CO_2_ [_45_]. Cells were coated with gold layer of 40 nm thickness. The SEM images were taken in Quanta^TM^ 250FEG imager (FEI GmbH, Frankfurt, Germany).

### Phylogenetic Analyses

The *P. judraskeda* full length rDNA operon was obtained by primer walking. Amplification was carried out with the combination of eukaryotic universal ribosomal primers and *Picomonas*-specific primers (1F -P01ITS1R; PICOBI01F-L52R; P01ITS1F-28S_1433rev; P01ITS2F-28S_1433rev; 28S_PicoD15F-28S_2933rev; 28S_PicoD21F-28S_2933rev; 28S_PicoG4For-28S 3356rev, Table S1). Overlapping primers were designed if necessary for the amplification. The detailed list of primers used in this study is given in Table S1. The complete nuclear ribosomal operon of *Picomonas judraskeda* has been deposited at NCBI Genbank with acc no JX988758. Furthermore, we constructed ‘(pico)biliphyte’-specific clone libraries as given in Medlin et al. 2006 [46], using taxon-specific primers (PICOBI01F and L52R, Table S1) to study the diversity of ‘(pico)biliphytes’ in the same habitat. In addition, one environmental sequence was obtained for clade ‘BP2’ from same habitat by PCR using PICOBI02F and L52R primers (Table S1).

Overall 191 environmental picozoan 18S rDNA sequences retrieved from the Genbank database (http://www.ncbi.nlm.nih.gov/genbank/; Accessed 2012 July), nine new environmental sequences (acc no. JX988759- JX988767), and the sequence (acc no JX988758) obtained from the cultivated strain *P. judraskeda* were subjected to phylogenetic analyses. Prior to the analyses, the manual alignment process was guided by the conserved secondary structure of the 18S rRNA obtained from other known eukaryotes [47] using SeaView 4.3.2 as an alignment editor. Sequence regions that had uncertain alignment positions were post-aligned by Mfold [48]. Because of some shorter environmental sequences (lacking the 5′-part of the gene), only 1253 (from a total of 1798 bp of the 18S rDNA from our strain) unambiguously aligned characters could be used for the phylogenetic analyses (the alignment is available upon request). Tree topology (maximum likelihood, ML) was calculated by using RAxML PTHREADS version 7.2.6 [49]. To determine the best tree topology, 20 ML trees were computed starting from 20 randomized maximum parsimony starting trees. The GTR+I+Γ evolutionary model was used and 1000 maximum likelihood bootstrap replicates were calculated by RAxML. In addition, PAUP 4.0 B10 [50] was applied to calculate 1000 Neighbour Joining (NJ) bootstrap replicates. For NJ, the GTR+I+Γ evolutionary model was selected by Modeltest v3.7 [51]. Bayesian interferences were calculated from MrBayes with the covarion option by using two runs with four Markov chain Monte Carlo (MCMC) chains over 3,000,000 generations, and the first 1,000,000 generations were discarded as “burn-in” [52], [53]. Bootstrap and Bayesian posterior probabilities values less than 60% and 0.80 respectively were considered as “negligible support.” Two taxon samples (MS584-5, MS584-11) were reassembled by contig assembly obtained from the Single-cell Amplified Genome data of Yoon et al. (2011). Accession numbers for all sequences are depicted on the tree (Fig. S2).

### Nomenclatural Acts

The electronic version of this document does not represent a published work according to the International Code of Zoological Nomenclature (ICZN), and hence the nomenclatural acts contained in the electronic version are not available under that Code from the electronic edition. Therefore, a separate edition of this document was produced by a method that assures numerous identical and durable copies, and those copies were simultaneously obtainable (from the publication date noted on the first page of this article) for the purpose of providing a public and permanent scientific record, in accordance with Article 8.1 of the Code. The separate print-only edition is available on request from PLoS by sending a request to PLoS ONE, 1160 Battery Street Suite 100, San Francisco, CA 94111, USA along with a cheque for $10 (to cover printing and postage) payable to “Public Library of Science.”

In addition, this published work and the nomenclatural acts it contains have been registered in ZooBank (http://zoobank.org/), the proposed online registration system for the ICZN. The ZooBank LSIDs (Life Science Identifiers) can be resolved and the associated information viewed through any standard web browser by appending the LSID to the prefix “http://zoobank.org/.” The LSID for this publication is: urn:lsid:zoobank.org:pub:009138ED-3F29-4351-894D-AD2E9CAC28D0.

## Results

### Isolation and Cultivation of *Picomonas judraskeda* sp. nov

Seawater samples collected between July and October in 2007, and August 2008 from Helgoland Roads (Germany) and subsequently filtered through 10 µm and 2 µm membrane filters, contained DNA from ‘(pico)biliphytes’ as demonstrated by PCR amplicons using primers specific for ‘(pico)biliphytes’ (results not shown). ‘(Pico)biliphyte’- positive samples were regularly transferred into 0.1 µm filter-sterilized seawater (FSSW) and the presence of ‘(pico)biliphytes’ monitored by PCR and DNA sequencing. These ‘enrichments’ were used for flow cytometry and sorting. We employed a mitochondrial marker (MitoTracker® Green FM) and obtained a single cell-derived culture verified by rDNA sequencing using ‘(pico)biliphyte’-specific and universal eukaryotic ribosomal DNA (rDNA) probes (Fig. 1). Investigation of the culture by light and electron microscopy verified that it contained only a single species of eukaryote, here described as *Picomonas judraskeda* gen. nov., sp. nov. for which we erect a new phylum (Picozoa phylum nov.). In addition, the culture contained several types of morphologically distinguishable bacteria. In general, we found that FSSW, with very low nutrient concentrations and the presence of bacteria (an axenic culture of *P. judraskeda* could not be established) were essential for maintaining successful growth, which was nevertheless slow and sometimes unpredictable, depending on the source of seawater (seawater from Helgoland, the Canary Islands and South Africa was tested), the season during which the seawater was collected (seawater from Helgoland Roads collected between October and June generally did not support growth of the organism), as well as on recovery from a lag phase of several days after serial transfer. The maximum cell density of *P. judraskeda* observed in culture was 30–40 cells/ml (cells were identified by phase contrast microscopy based on their peculiar swimming behavior while focusing through the whole water column of the tissue flask). The organism was maintained for 3 years in culture until it died in June 2012 after unsuccessful serial transfers.

### Light Microscopy and Cell Movement

**Figure 2:**
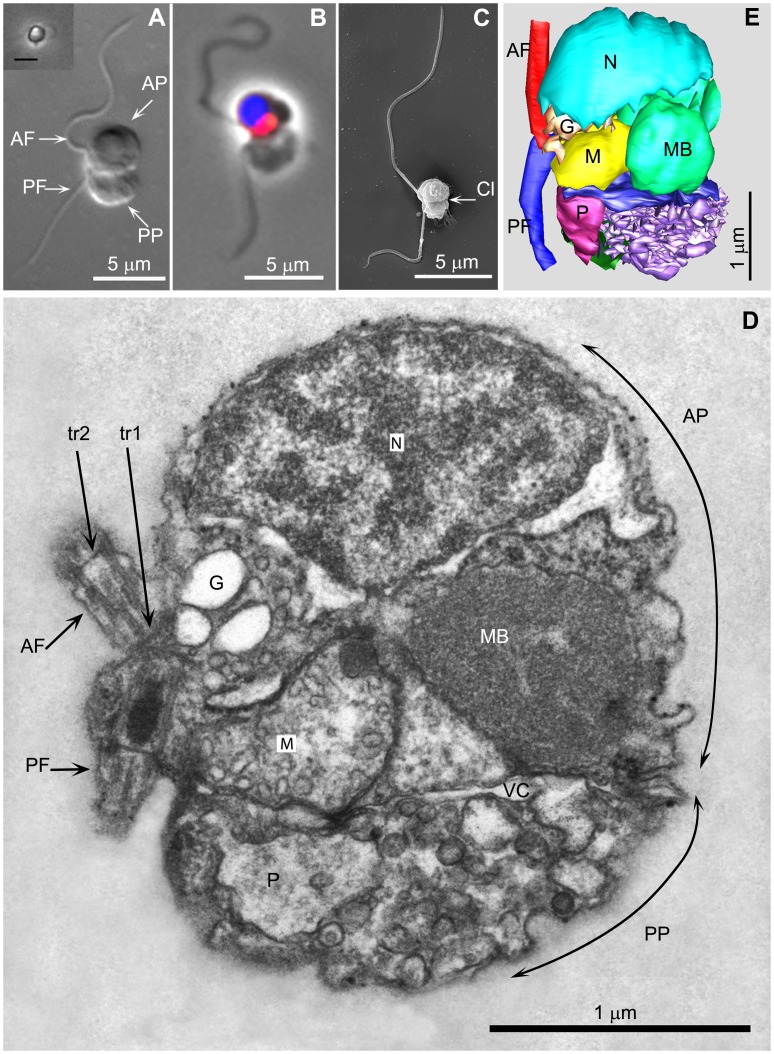
A *Picomonas* cell. 2A. Differential interference contrast of a chemically fixed cell. Inset shows phase contrast image of a live cell from tissue culture flask photographed with an inverted microscope (Scale bar 5 µm). 2B. Fluorescence and phase contrast overlay, nucleus (blue), mitochondrion (red). 2C. SEM image. 2D. A longitudinal section through a cell in the plane of the flagella, viewed from the cell’s left. 2E. A 3 D serial section reconstruction of the cell depicted in 2D. AF/PF (anterior−/posterior flagellum); AP/PP (anterior/posterior part of the cell); G (Golgi body); M (mitochondrion); MB (‘microbody’); N (nucleus); tr_1_,tr_2_ (distal [tr2] and proximal [tr1] flagellar transitional regions); P (posterior digestive body); Cl (cleft separating the anterior from the posterior part of the cell); vc (vacuolar cisterna).

The cells of *P. judraskeda* sp. nov. are mostly stationary, floating in the water column. The oblong cells vary in length between 2.5–3.8 µm, their width being 2–2.5 µm (n = 20). Larger cells (lengths >4 µm and widths >3 µm) were only observed by LM in chemically fixed preparations (Fig. 2A,B). The cells are biflagellate with a long (12–14 µm) and a short (7–9 µm) flagellum inserted laterally near the middle of one of the long sides of the cell (Fig. 2A-C). This cell side is termed ventral, the opposite dorsal. The long flagellum is arbitrarily termed the anterior flagellum, the short flagellum the posterior flagellum. Each cell consists of two nearly hemi-spherical parts separated by a deep cleft (Cl, Fig. 2C). The anterior part (AP) contains the nucleus and the single mitochondrion (Fig. 2B), whereas the posterior part (PP) varies in size (presumably depending on the trophic status of the cell or the stage of the feeding cycle, see below), which accounts for the observed relatively large variations in cell length.

The cells of *P. judraskeda* exhibit a unique mode of motility: After extended periods of rest, a stereotypic pattern is initiated consisting of a rapid short-distance jump of the cell into the anterior direction (approximately 3–5 µm), immediately followed by a slower dragging cell movement in the opposite (posterior) direction. The drag movement lasted for about 2s, during which the cells travelled a distance of approximately four times their cell length. This jump/drag- cycle was observed from two up to five times consecutively after which the cells suddenly ‘shot’ away (a behavior termed ‘skedaddle’ here, a colloquial term meaning to move away rapidly). During skedaddling, the cell travelled a distance of minimum 50 µm before it became immotile again and until the next jump/drag cycle was observed (Movie S1). During resting periods of the cell, the posterior flagellum (PF) was held closely adjacent to the ventral cell surface curving around the posterior end of the cell, often visible under the microscope and mostly immotile, while the anterior flagellum extended from the cell surface revealing an undulatory wave pattern. The behavior of the flagella during the jump/drag/skedaddle cycle was not studied in detail but apparently involved flagellar re-orientation and rapid undulatory movements of one or both flagella. Occasionally late stages of cell division have been observed; the daughter cells violently separate from each other using the skedaddle type movement for cell separation (not shown).

### Electron Microscopy

The ultrastructure of *P. judraskeda* has revealed several highly unusual features that collectively, to the best of our knowledge, have not been described before in any other eukaryotic cell. The cell is distinctly separated into two parts. The anterior part (AP) contains almost all cell constituents of a typical eukaryote cell: The large hemispherical nucleus occupies nearly half the volume of the anterior cell part and in its spherical part, the nuclear envelope is appressed to the plasma membrane (Fig. 2D,E; Fig. S1; Movie S2). The interphase stage of the nucleus appears to have a considerable amount of condensed chromatin, which is uncommon in unicellular organisms.

**Figure 3:**
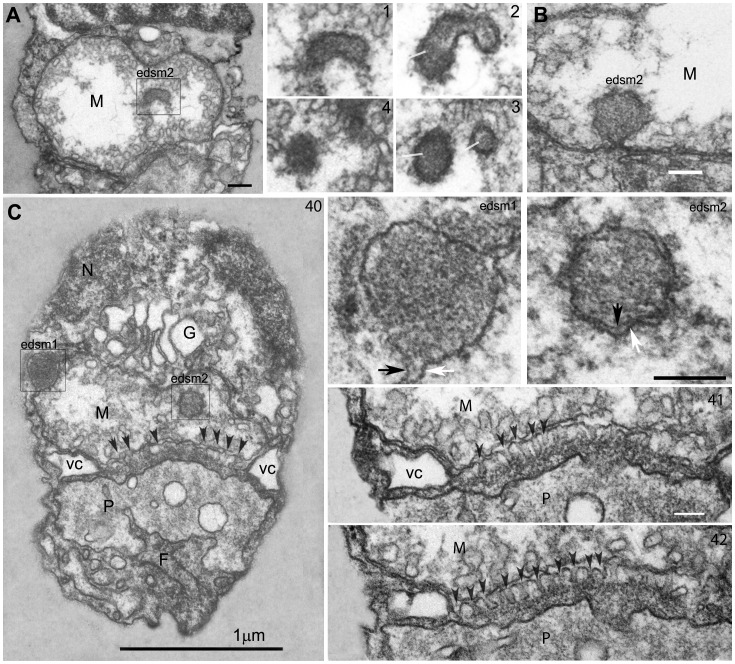
Ultrastructure of the mitochondrion of a *Picomonas* cell. Single mitochondrion with tubular cristae, membrane bound (single and double) electron dense material (edsm1 and 2 respectively) and regular projections of the outer envelope membrane. 3A. a double membrane bound edsm2 near the ventral surface; serial sections (1–4) of the edsm2 displayed some tube-like structures in the lumen (thin lines); also note that the edsm2 is a branched structure. 3B. A membrane invagination of edsm2 into the mitochondrion that reveals continuity between the mitochondrial envelope membranes and the two membranes encircling the edsm2. 3C. Oblique section of a cell from dorsal right to ventral left with edsm1 and edsm2 displayed. The edsm1 (on the left) is positioned between the outer (black arrow) and inner (white arrow) mitochondrial envelope membrane and the edsm2 (on the right) with a double membrane (higher magnifications of the two edsms on the right. A large number of short cylindrical membrane protrusions termed ‘mitovilli’ (arrow heads) extend from the outer mitochondrial membrane towards posterior digestive body (P), they terminate in granular material covering the posterior digestive body. A vacuolar cisterna (vc) in the ‘cleft’ region separates the anterior from posterior part of the cell and is only absent (i.e. contains a large hole) in the area of the mitovilli (3C). Towards the right are shown higher magnifications of two serial sections from the cell depicted in 3C revealing details of the mitovilli-posterior digestive body junction. F (feeding apparatus with basket fibers; G (Golgi body); M (mitochondrion); N (nucleus); P (posterior digestive body); vc (vacuolar cisterna). Numbers at the top right indicate the section number of a series. Scale bar: 100 nm.

The single mitochondrion with tubular cristae (Fig. 3A,C) is located posterior to the nucleus in the ventral region of the anterior part (Fig. 2D,E; Movie S2). Its shape resembles that of a sofa/couch that extends from near the left to the right surface of the cell (length ∼1.6 µm, Fig. 3A), with its high back (height ∼ 1 µm) oriented towards the cell’s center, the end touching the nuclear envelope (Fig. 2D). The seating area narrows towards the ventral region (Fig. 2D) and terminates near the ventral surface at the level of the basal body of the posterior flagellum (Fig. 2D,E). The mitochondrion displays two additional highly distinctive structural differentiations:

1. There are two membrane-bound inclusions with electron-dense, granular contents located at specific positions inside the mitochondrion (edsm1, edsm2, Fig. 3). The first (edsm1) is located at the high back end of the mitochondrion, positioned within the inter-membrane space which is thereby dilated (Fig. 2D; Fig. 3C, inset). Appropriately sectioned, this inclusion appeared to be located in the matrix enclosed by a single membrane (the inner envelope membrane). Because the edsm1 could be followed through 7 serial sections (a cell longitudinally sectioned from left to right in Fig. S1), we assume that the shape of this inclusion is cylindrical (diameter 150–200 nm, length minimum 400 nm). The second (edsm2) is located in the ventral, left part of the mitochondrion and is enclosed by two membranes (Fig. 3A,C, inset). This inclusion also has contact with the mitochondrial envelope and in one fortuitous section, we were able to demonstrate the continuity between the two membranes enclosing the edsm2 and the mitochondrial envelope (Fig. 3B), i.e. the edsm2 is a tubular invagination of the cytoplasm into the mitochondrial matrix. Serial sections revealed that the edsm2 is a branched structure; its contents sometimes appeared to contain small tubules rather than granules as in the edsm1 (Fig. 3A, sections 1–4).
2. The lower side of the mitochondrion adjacent to the posterior part of the cell (the bottom of the seating area) displayed another highly unusual specialization. Over an area of ∼ 1×0.6 µm (Fig. 3A,C; Movie S2), regularly-spaced projections extend from the outer mitochondrial envelope membrane towards the posterior part (PP) for 50–60 nm (n = 30) after which they terminate in an electron dense granular area that is in contact with the membrane of a large vacuole (the posterior digestive body, see below). These projections, termed here “mitovilli,” are about 20 nm wide (narrower near their base) and are spaced at 40 nm (Fig. 3C, sections 41, 42). From this we calculate that the specialized surface of the mitochondrion displays about 300 mitovilli (Movie S2).

**Figure 4:**
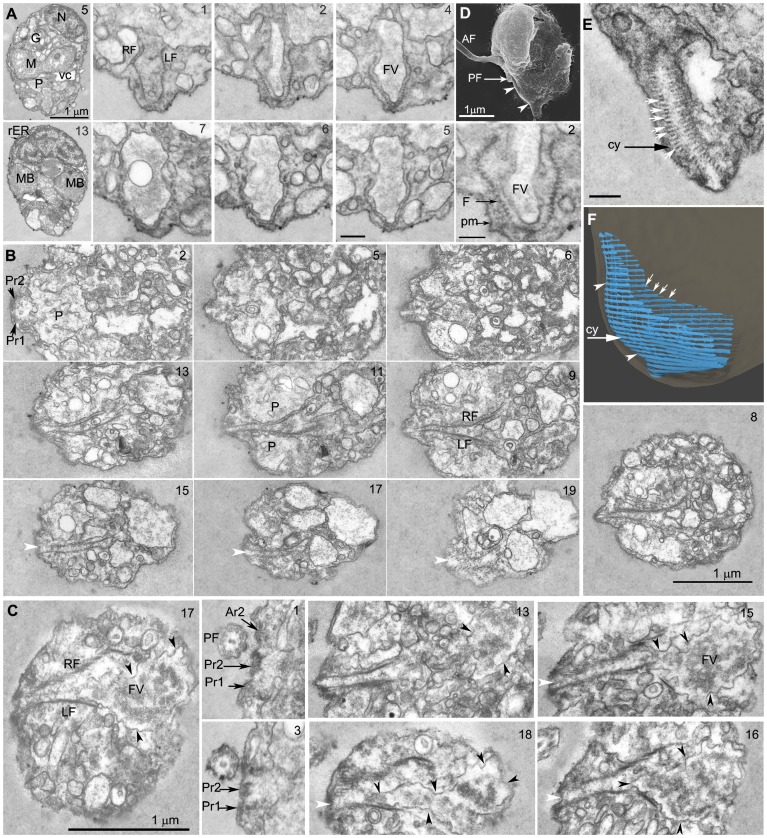
Feeding apparatus. 4A. Longitudinal sections of a *Picomonas* cell from ventral to dorsal with the feeding apparatus in cross section. Sections 2 to section 7 depict a recently formed food vacuole inside the ‘basket’ of the feeding apparatus. 4B & C. Cross sections through two cells of *Picomonas* from anterior to posterior; sections begin in the central part of the cell, posterior to the basal bodies. 4B. Cell, in the non-feeding mode, without a food vacuole within the basket. 4C. Cell during active feeding with a large food vacuole within the basket (black triangles); the food vacuole contains irregularly-shaped, ‘fuzzy’ material, presumably taken up by endocytosis through the cytostome (white triangles). Two rows of fibers representing the left (LF) and right (RF) margins of the basket accompany the food vacuole. 4D. An SEM image of *Picomonas* visualizing the left side of the cell. Note that the posterior flagellum has been shed at the tr2; white triangles indicate the cytostome region of the feeding apparatus. 4E. Longitudinal section through the basket near the cytostome. It shows approx. 60 rows of fibers arranged in parallel (white arrows depict fibers in the right margin of the basket). 4F. 3D model of the feeding apparatus with rows of fibers in parallel arrangement (white arrows) forming a basket (arrangement of the cell as in 4D). Note that the fibers are interconnected by thin filaments. The basket is open towards the top and right (i.e. towards the anterior and dorsal direction of the cell respectively), while it is closed at the bottom (the posterior end of the cell). On the left side of the basket (representing the cell’s ventral surface) the fibers are attached to the plasma membrane thus forming the narrow, slit-like cytostome. AF/PF (anterior−/posterior flagellum); G (Golgi body); LF/RF (left and right row of fibers of the basket); M (mitochondrion); MB (microbody); N (nucleus); P (posterior digestive body); Ar2 (anterior microtubular flagellar root 2); Pr1 (posterior microtubular flagellar root 1); Pr2 (posterior microtubular flagellar root 2); FV, (food vacuole); vc (vacuolar cisterna); cy (cytostome). Numbers at the top right indicate the serial section. Scale bar: 200 nm; except Fig. 4A, section 2 (100 nm).

The single Golgi body is located between mitochondrion, nucleus and basal body of the anterior flagellum closely associated (resting on) with the seating area of the mitochondrion (Fig. 2D,E; Fig. 3C; Movie S2). It consists of 5–7 Golgi cisternae (often artificially inflated caused by suboptimal chemical fixation), the cis-face oriented towards the nucleus, the trans-face towards the basal body. Two sheets of rough endoplasmic reticulum extend from the nuclear envelope posteriorly along the left and right sides of the anterior part of the cell, respectively (Fig. 4A section 13; Movie S2).

Two large, spherical microbodies (MB; with granular contents, bounded by a single membrane) of 600–800 nm diameter are located in the dorsal region of the anterior part of the cell, besides the mitochondrion (Fig. 2D,E; Fig. 4A; Fig. S1, sections 26, 29, 32; Movie S2).

The anterior part of the cell is separated from the posterior part by a large, horizontally-oriented, plate-like vacuolar cisterna (vc, Fig. 2D,E; Fig. 3C; Fig. S1; Movie S2), that was sometimes observed to be dilated (Fig. S1, likely an artifact of chemical fixation). At its margin, the vacuolar cisterna is appressed to the plasma membrane (Fig. 3C; Fig. S1), except near the ventral cell surface where space is left for passage of three microtubular flagellar roots into the posterior part of the cell (see below (Fig. 6; Fig. 7)). The vacuolar cisterna displays a large hole in the region where the mitovilli abut the posterior part of the cell (Fig. 3C sections 41, 42; Fig. S1 sections 9–15).

The posterior part of the cell consists of numerous vesicles and vacuoles of different sizes and electron density but lacks ribosomes and endoplasmic reticulum, which are confined to the anterior part. In addition it contains the cytostome and feeding basket (see below). The lengths (and volumes) of the posterior part (PP) were found to vary considerably among sectioned cells (from anterior to posterior: 0.6–1.5 µm, compare e.g. Fig. 2 with Fig. S1, section 13), whereas the lengths of the anterior part (AP) of the cells were quite constant (1.9–2 µm, both n = 10). Two regular structures were identified in the posterior part of the cell:

The first structure is a large vacuole (“posterior digestive body,” P) that is located in the ventral region of the posterior part of the cell adjacent to the vacuolar cisterna and the mitovilli (Fig. 2D,E; Fig. 3C; Movie S2). Its shape is basically a short bar with the long axis extending from the left to the right cell side (∼ 1 µm; Fig. 4A,B), its depth (from ventral to dorsal) 600–700 nm and its height 400–500 nm. The membrane surface of this vacuole is often highly irregular with invaginations (Fig. 3C). Sometimes these invaginations contain vesicles (Fig. 4B, section 5). The contents of this vacuole consist of irregular, fluffy material of medium electron density and numerous vesicles of various sizes with electron-translucent contents (the occasional denser vesicles/tubules presumably represent the invaginations described above, Fig. 2D; Fig. 3C).

The second conspicuous structure in the posterior part (PP) of the cell is the cytostome/feeding basket that together comprise the feeding apparatus (‘F’, Fig. 4). The feeding apparatus is located ventro- posterior to the posterior digestive body (P) and consists of a basket of about 50–60 parallel running fibers (diameter of a single fiber: 15 nm, 30 nm repeat structure from one fiber to the next) of varying lengths that extend perpendicular to the anterio-posterior cell axis, from the ventral surface where they are attached to the plasma membrane for up to 1.2 µm enclosing the cytostome, towards the dorsal region where they terminate (Fig. 4B,C,F; Movie S2; Movie S3). In addition to these major fibers, there is a fine network of very thin fibers that interconnect adjacent fibers irregularly (not shown; Fig. 4F). The basket is open towards the anterior and dorsal regions of the posterior part (PP), but closed towards the posterior end of the cell (Fig. 4A–C) where it sometimes extends into a short tail-like projection of the cell (Fig. 4A section 1 and Fig. 4D,E). Towards the dorsal end, the feeding basket gradually widens (up to 500 nm at its dorsal end). The slit-like cytostome (‘cy’, see Fig. 6C) is formed where the ‘side walls’ of the basket fibers connect to the plasma membrane on the ventral surface (the fibers are anchored in electron-dense ribbons at the plasma membrane; Fig. 4C, sections 13, 15–18; white arrow head) and extends in the anterior-posterior direction for a length of about 1 µm, its width being on average 150 nm (n = 10; Fig. 4B–E; Movie S2; Movie S3). In serial sections of some cells it was observed that the anterior-most part of the feeding basket bisected the overlying posterior digestive body (Fig. 4B, section 11). In these cells (non-feeding stage), the feeding basket appeared to contain mostly small vesicles and multivesicular bodies (Fig. 4B). In other cells, possibly during active feeding, the feeding basket contained a single, large vacuole that extended well beyond the basket into the dorsal region of the posterior part (PP) to reach the dorsal plasma membrane (Fig. 4C, sections 15–18; the length of this vacuole could be up to 1.2 µm). This vacuole contained irregular-shaped, fluffy material of medium electron density (very rarely a vesicle was seen within this vacuole; Fig. 4A, section 7). We term this vacuole the “food vacuole (FV).” The food vacuole extended ventrally into the narrow parts of the basket (Fig. 4C, sections 16–18), where it was sometimes observed to be associated with smaller vesicles (Fig. 4C, section 17). The anterior end of the cytostome (and thus the feeding basket at the plasma membrane) is linked to the posterior ends of two microtubular flagellar roots (Pr1, Pr2, see below for description of the flagellar apparatus; Fig. 4B, section 2 and Fig. 4C, sections 1, 3; Movie S2) through the two electron-dense ribbons attached to the plasma membrane that mediate this connection. We also noted that the posterior flagellum is located along the ventral cell surface in close proximity to the cytostome (200–400 nm distance) running almost parallel to the cytostome slit (Fig. 2E; Fig. 4C, sections 1,3,16; Fig. S1, section 7; Movie S2).

**Figure 5:**
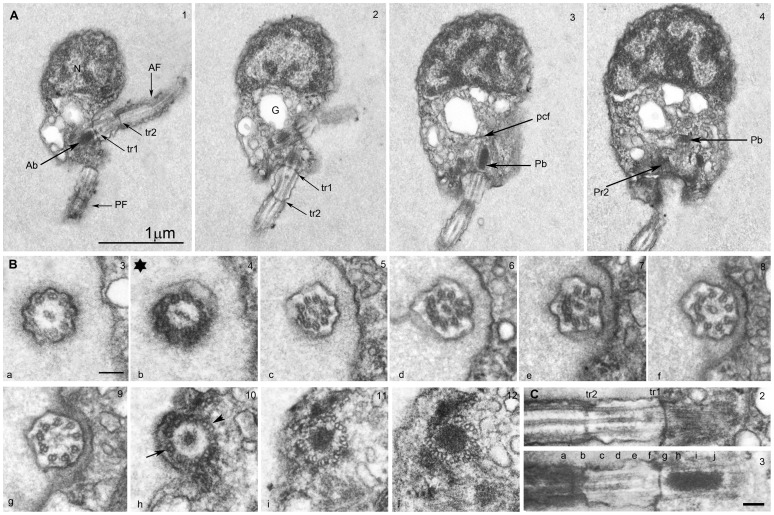
Flagellar apparatus. 5A. Longitidinal section of *Picomonas* from ventral to dorsal (sections are oblique from anterior part). Anterior flagellum (AF) and posterior flagellum (PF). The anterior flagellum locates close to the ventral surface followed more interiorly by the posterior flagellum. The basal bodies of both flagella are connected by a proximal connecting fiber (pcf) and exhibit two transitional regions (the proximal is termed ‘tr1’ and the distal ‘tr2’). 5B, C. Consecutive serial cross sections and two serial longitudinal sections of the anterior flagellum. Serial sections of 5B correspond to 5C, denoted in 5B_a-j at the left lower end. a. The axoneme with 9outer doublet and 2 central pair microtubules. b. The distal trasitional region (tr2) is involved in flagellar shedding (* indicates electron dense material near outer doublets). f. the central pair of microtubules orginate. h. the transitional region 1 (tr1), C-tubules added (arrowhead indicates a microtubulular triplet and arrow indicates a microtubular doublet. i,j. cross sections through basal body with microtubular triplets arranged in the clockwise direction, the basal body lumen is filled with electron dense material. AF/PF (anterior−/posterior flagellum); tr_1_, tr_2_ (distal [tr2] and proximal [tr1] flagellar transitional regions); Ab/Pb (anterior−/posterior basal body); pcf (proximal connecting fiber); G (Golgi body); N (nucleus); Pr2 (posterior microtubular flagellar root 2). Numbers at the top right indicate the serial section. Scale bar: 100 nm.

**Figure 6:**
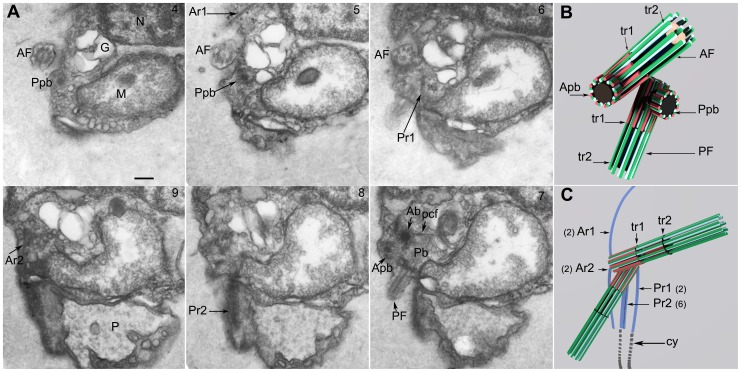
Basal apparatus and probasal bodies. 6A. Electron micrograph shows consecutive serial sections of *Picomonas judraskeda* from left to right (Sections are angled about 30 degree towards left from central axis). Each probasal body diverges from its parental basal body at right angles, both probasal bodies are oriented towards the ventral surface of the cell and are attached to the plasma membrane. 6B. 3D- scheme of the arrangement of basal bodies with probasal bodies. 6C. A schematic presentation of the flagellar basal apparatus seen from the ventral surface of the cell with proximal parts of the axonemes, basal bodies and four microtubular flagellar roots (for details see text). Numbers in brackets indicate the number of microtubules in each root. AF/PF (anterior−/posterior flagellum); tr_1_,tr_2_ (distal [tr2] and proximal [tr1] flagellar transitional regions); Ab/Pb (anterior−/posterior basal body); Apb/Ppb (anterior/posterior probasal body); pcf (proximal connecting fiber); Ar1/Ar2 (Anterior microtubular flagellar roots 1 and 2); Pr1/Pr2 (posterior microtubular flagellar roots 1 and 2); G (Golgi body); N (nucleus); M (mitochondrion); cy (cytostome); P (posterior digestive body). Numbers at the top right indicate the number of the serial section. Scale bar: 200 nm.

The two flagella emerge from the ventral cell surface of the cell, close to the junction of the Golgi body and the ventral tip of the mitochondrion (Fig. 5A; Fig. S1; Movie S2). Both flagella have a smooth surface and lack appendages (hairs, scales) when observed by TEM and SEM (Fig. 5A,B; see also Fig. 2C). Their tips form short hair-points of on average 0.6 µm length (n = 5; not shown). The flagellar bases form an angle of 120–140° (n = 5). When viewed from the ventral side, the anterior flagellum projects towards the cell’s left, its base forming an angle of about 40° to the anterio-posterior cell axis (Fig. 5A; Fig. 6C; Movie S2). The posterior flagellum deviates only slightly (∼10–20°) from this axis projecting towards the cell’s right and extending to the cell’s posterior. The two basal bodies are displaced against each other by about one basal body diameter, in that the anterior basal body is located more ventrally than the posterior basal body (Fig. 5A; Fig. 6A,C; Movie S2). Except for a single proximal connecting fiber (Fig. 5A, section 3) both basal bodies were not interconnected by fibrous structures.

**Figure 7:**
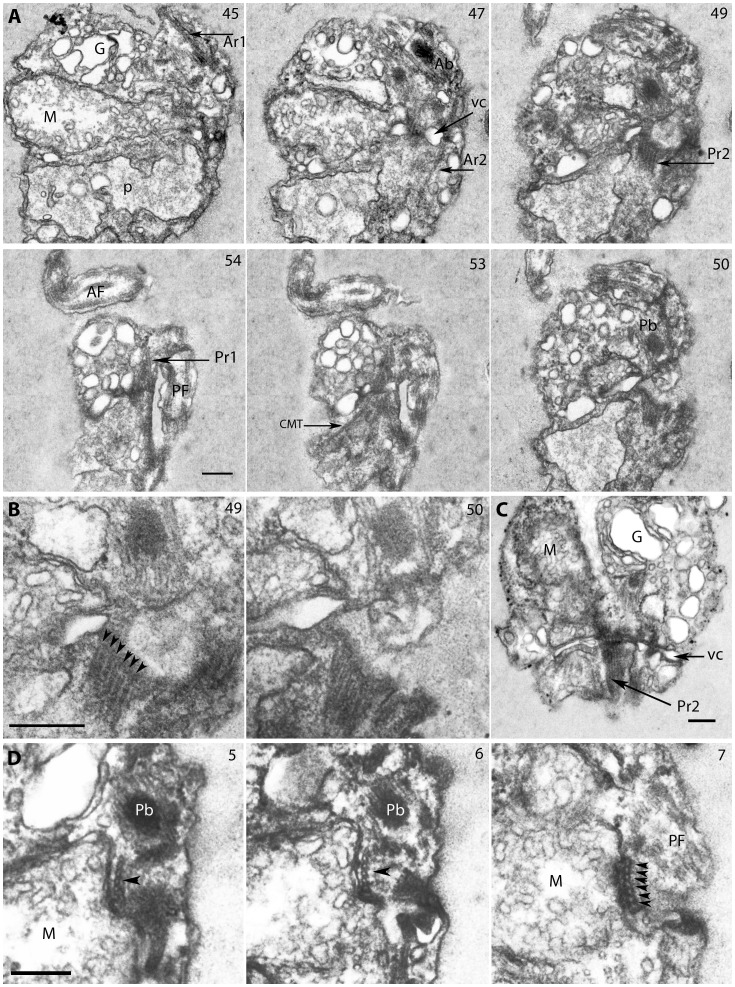
Flagellar root system in *Picomonas judraskeda*. 7A. Non-consecutive serial sections from dorsal to ventral (sections are oblique to cell’s right). Each basal body is connected to two microtubular flagellar roots. The anterior root 1 (Ar1) runs anteriorly to cell’s left. The Ar2 runs posteriorly, at the right side of the cell and passes the cleft (cl). The other two flagellar roots originate from the posterior basal body and extend towards the posterior part of the cell. One of the posterior flagellar roots (Pr1) runs on the left side of the cell. The other broader posterior flagellar root (Pr2), runs between the Ar1 and Pr1. 7B. The Pr2 with 6 microtubules (arrowheads) obliquely sectioned. 7C. A cell with the Pr2 passing the vacuolar cisterna and mitochondrion. 7D. Consecutive serial cross sections through the Pr2 located in a depression of the mitochondrion. AF/PF (anterior−/posterior flagellum); Ab/Pb (anterior−/posterior basal body); Ar1/Ar2 (Anterior microtubular flagellar roots 1 and 2); Pr1/Pr2 (posterior microtubular flagellar roots 1 and 2); G (Golgi body); M (mitochondrion); vc (vacuolar cisterna); P (posterior digestive body); CMT (secondary cytoplasmic microtubule). Numbers at the top right indicate the number of the serial section. Scale bar: 200 nm.

The basal body and the flagellar transitional region display several unusual features: Basal bodies are relatively short (360–380 nm) and most of their lumen is filled with electron dense material (Fig. 5A,B). This material seems to have thin connections to the A-tubules of the microtubular triplets (Fig. 5B, section 11) and to the proximal transitional plate (Fig. 5C, section 3). Often the electron dense material consists of a longer, distal part of high electron density (260–290 nm in length) and a shorter proximal part of lower electron density (60–80 nm in length), separated by an electron- translucent space of about 30–50 nm in length (Fig. 5A, sections 2, 3). A cartwheel has not been observed in either of the basal bodies (but may have been obscured by the proximal electron dense material). Another unusual feature of the flagellar bases is the presence of two transitional plates in the proximal part of each flagellum (Fig. 5A,C, (tr1& tr2)). The spacing between the transitional plates was usually around 380–400 nm but occasionally could be as short as 320 nm (Fig. 5A,C). Both transverse plates appeared to be structurally identical, however, in our fixation, the distal transverse plate (tr2) constricted the flagellum and axoneme (Fig. 5A,C). At this site the flagellum seems to be shed because we often observed flagellar stumps terminating with the tr2 (see also Fig. 4D, PF; Fig. 6A section 7). Interestingly, the central pair of microtubules originate near the tr1 and extend through the tr2 (Fig. 5A, section 2 and Fig. 5C, section 2).

During interphase, the basal bodies generally do not appear to be associated with probasal bodies, but in one cell (likely in preparation for cell division), we observed probasal bodies associated with each basal body (Fig. 6A,B, (Ppb, Apb)). Both probasal bodies extended from their parental basal bodies at right angles, and interestingly were oriented towards the same side, closely associated with the ventral surface of the cell (Fig. 6A, B).

Serial sections revealed the presence of four microtubular flagellar roots, two associated with each basal body. We have given the roots descriptive terms (Ar1, Ar2, Pr1, Pr2; Fig. 6C; Movie S2), because we did not study basal body development during the cell cycle. The Ar1 originates at the anterior basal body, consists of two microtubules and runs towards the cell’s anterior left (Fig. 6A, section 5; see also Fig. 7A, section 45). It accompanies the nuclear envelope and terminates before reaching the anterior end of the cell (not shown; Movie S2). The Ar2 originates at the anterior basal body (near its proximal end; Fig. 7A, section 47) and also consists of two microtubules, runs beneath the ventral cell surface to the cell’s posterior passing the mitochondrion and entering the posterior part (PP) of the cell, where it terminates near to the anterior tip of the cytostome just opposite to the posterior flagellum (Fig. 4C, section 1; Fig. 6C; Fig. 7A, section 47; Movie S2). The Pr1 originates at the left surface of the posterior basal body near its proximal end (Fig. 6A, sections 6, 7), consists of two microtubules, runs along the ventral surface of the cell to the cell’s posterior, passes the ventral surface of the mitochondrion, and enters the posterior part (PP) of the cell where it terminates near the anterior edge of the left side wall of the cytostome (Fig. 4B, section 2 and Fig. 4C sections 1, 3; Fig. 6A, section 6 and Fig. 6C; Fig. 7A, section 54; Movie S2). The Pr2 originates near the right surface of the posterior basal body (Fig. 6A, section 8 and Fig. 6C; Fig. 7A, sections 47, 49). It is a broad root consisting of 6 microtubules (Fig. 7B, sections 49, 50, and Fig. 7D, sections 5–7; Movie S2). It runs close to the ventral surface of the cell towards the cell’s posterior, passes the ventral surface of the mitochondrion, is located in a shallow depression of the latter (Fig. 7D, sections 5, 6), then traverses the vacuolar cisterna (Fig. 7C). The Pr2 takes a path parallel to the Pr1 extending into the posterior part (PP) of the cell (Fig. 6A, section 8) and terminates near the anterior end of the cytostome near its right side wall (Fig. 4B, section 2 and Fig. 4C, sections 1, 3; Fig. 6C; Movie S2). All three posteriorly oriented roots (Ar2, Pr1 and Pr2) run parallel to each other into the posterior part (PP) of the cell, spaced 0.5 µm (from Ar2 to Pr1; Fig. 4C, section 1; Fig. 6C; Movie S2). The Pr1 and Pr2 run for about 400 nm into the posterior part of the cell, where the microtubules terminate near the plasma membrane (the Ar2 seems to terminate before the other roots). At this position, electron dense ribbons associated with the plasma membrane extend from the Pr1 and Pr2 and continue alongside the left (from Pr1) and right (from Pr2) side wall of the cytostome to the cell’s posterior end (Fig. 4B,C; Fig. 6C; Movie S2). A few additional cytoplasmic microtubules (CMT in Fig. 7A, section 53) originate at an angle of about 45° from the proximal region of the Pr1, to extend to the cell’s left, and run for an unknown distance along the left surface of the anterior part of the cell close to the vacuolar cisterna (not shown).

The cells are covered only by the plasma membrane with no scales or glycocalyx being discernible. Often, rod-shaped bacteria were encountered apparently physically attached to the plasma membrane by their ends. These bacteria were (ultra)structurally intact and could associate with any part of the plasma membrane (except flagella) (not shown).

### Molecular Phylogeny

**Figure 8:**
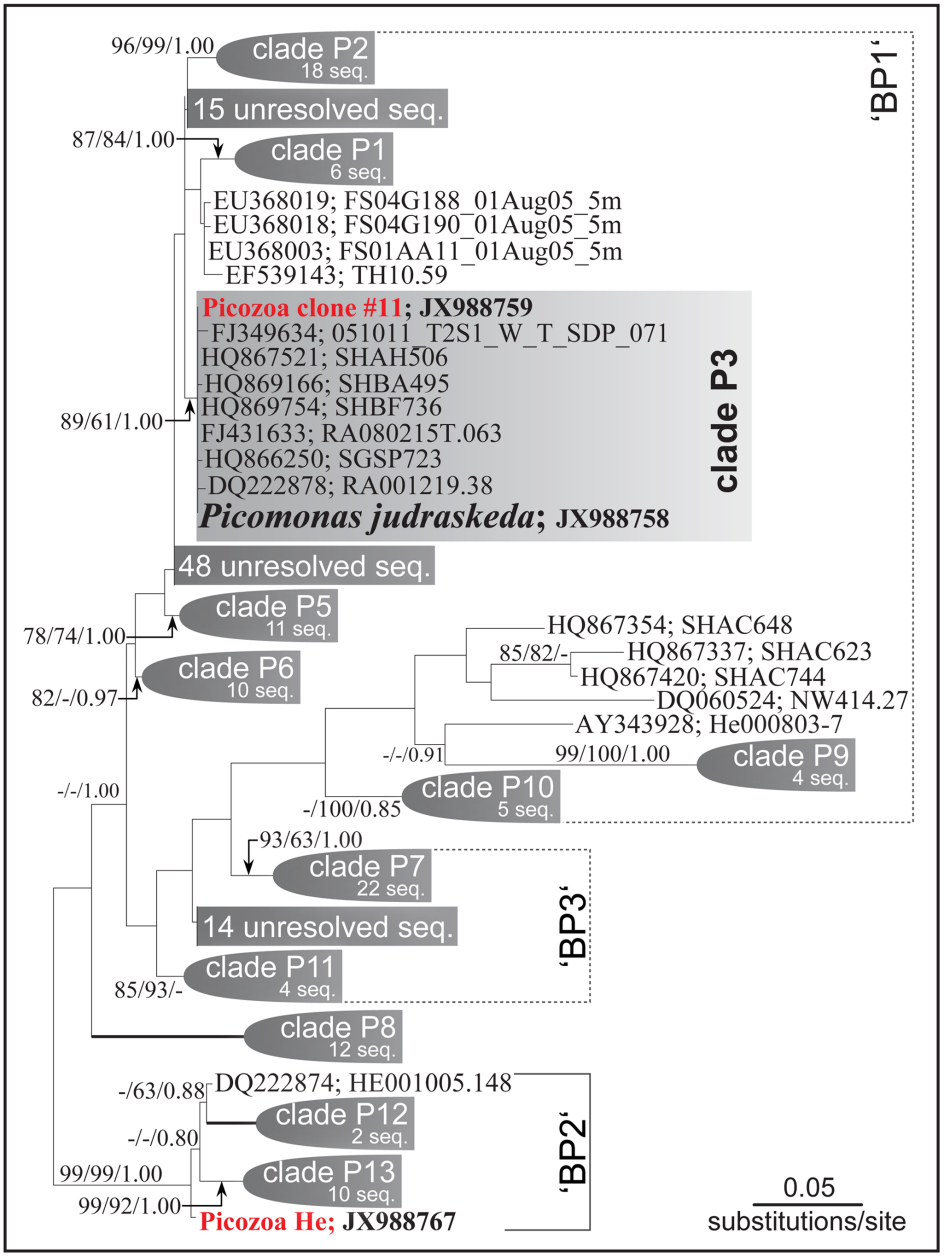
Unrooted randomized accelerated maximum likelihood (RAxML) phylogenetic tree of partial nuclear SSU rDNA of Picozoa. The phylogeny is based on 1253 aligned characters of the SSU rDNA and includes 201 sequences of Picozoa. Most sequences are database entries derived from clone libraries (nine environmental sequences generated from a sample taken at Helgoland Roads and one sequence from *Picomonas judraskeda* are new sequences; accession numbers of the newly determined sequences are presented in Fig. S2). Bootstrap values >60% and posterior probabilities >0.80 for the three methods of analyses used (RAxML/NJ by PAUP/MrBayes) are shown on the respective branches. Branches in bold show maximal (100%/1.00) support. Labeling of clades (‘BP1–3′) followed [Cuvelier et al. 2008], 12 novel clades (‘P1–P13’) are recognized (for details see Results). The sequence of *Picomonas judraskeda* (in bold) was positioned in clade ‘P3’ (shaded).

A molecular phylogenetic analysis of a broadly sampled taxon set (104 taxa of eukaryotes, excluding only Excavata) using a data set consisting of 18S rDNA, 5.8S rDNA and 28S rDNA (4461 aligned characters) could not position the Picozoa in one of the known eukaryotic supergroups. Neither monophyly of the Hacrobia [54] nor the previously reported sister group relation of Picozoa with the telonemids [41] could be substantiated in this analysis (results not shown). To explore the genetic diversity of the Picozoa, phylogenetic analyses of 201 partial nuclear-encoded SSU rDNA sequences of Picozoa (with the exception of *P. judraskeda* all others derived from environmental clone libraries) were performed (Fig. 8). The tree revealed a high genetic diversity within the Picozoa (for a tree with all accessions, see Fig. S2). Of the three major clades previously reported in the Picozoa (‘biliphyte’ clades ‘BP 1–3’; [36]) only one (‘BP2’) was monophyletic in our analyses. We tentatively identify 12 novel clades (‘P1–P13’, for Picozoa clades 1–13) in the Picozoa (clade P4 positioned within the “15 unresolved taxa” in ‘BP1’ is currently uncertain and not depicted in Fig. 8) that were well-supported by bootstrap and Bayesian posterior probability values (except for clades 10 and 11; Fig. 8). In addition to clades 1–13, many sequences remained within unresolved assemblages, presumably because of the low number of informative sites available in these partial SSU rDNA sequences. *Picomonas judraskeda* was positioned in the well-supported clade P3 whose members had almost identical sequences and included also “clone #11” from our environmental clone library of the same habitat (Fig. 8). Seven other of our environmental clones of Picozoa were positioned in clade P2, whereas one clone (“Picozoa He”) was a new genotype in clade ‘BP2’ (Fig. 8).

### Taxonomic Summary

### Phylum Picozoa phylum nov

#### Diagnosis

Heterotrophic, marine protists of picoplanktonic size (cells may pass through a 3 µm membrane filter) mostly characterized by either of two signature sequences in the nuclear-encoded SSU rDNA, 5′**GC**G **TG**A **TGC CAA AAT CCG**3′ (PICOBI01) or 5′**AT**A T**GC CCG TCA AAC CGT**3′ (PICOBI02).

### Class Picomonadea class nov

#### Diagnosis

With the characteristics of the phylum.

### Order Picomonadida ord. nov

#### Diagnosis

With the characters of the phylum. Taxa are characterized by the signature sequence 5′**GC**G **TG**A **TGC** **CAA** **AAT** **CCG**3′ (PICOBI01) that represents a molecular synapomorphy of the order. The most inclusive clade containing taxa with the sequence accession numbers GU822951 (clade P6), HQ865255 (clade P5) and *Picomonas judraskeda* (JX988758; clade P3), but not HQ868810 (clade P8) and JX988767 (‘BP2’). This is a branch-based definition in which all of the specifiers are extant. The Picomonadida includes at least clades P1–P6 plus several hitherto unresolved taxa.

### Family Picomonadidae fam. nov

#### Diagnosis

With the characters of the order. The most inclusive clade containing taxa with the sequence accession numbers HQ868687 (unresolved), EU368015 (clade P1), EU368029 (unresolved), DQ222877 (clade P2) and *Picomonas judraskeda* (JX988758; clade P3), but not GU822951 (clade P6) and HQ865255 (clade P5). This is a branch-based definition in which all of the specifiers are extant. The Picomonadidae includes at least clades P1–P3 plus several hitherto unresolved taxa.

### Type Genus. *Picomonas* gen. nov

### Genus *Picomonas* gen. nov

#### Diagnosis

Cells are biflagellate with a long and a short flagellum inserted laterally. Each cell consists of two nearly hemi-spherical parts separated by a cleft. The anterior part contains the flagellar apparatus, nucleus, endoplasmic reticulum, single Golgi body and mitochondrion with tubular cristae, whereas the posterior part is variable in size and contains the feeding apparatus and numerous vesicles and vacuoles. The anterior and posterior parts of the cell are separated by a single, large, vacuolar cisterna that leaves only part of the mitochondrion in direct contact with the posterior part. The cells exhibit a unique mode of motility: After extended periods of rest, a stereotypic pattern is initiated consisting of a rapid, short-distance jump, immediately followed by a slower, dragging cell movement in the opposite direction. This pattern may be repeated several times before cells finally show an extremely fast and extended movement away from their original position (termed ‘skedaddle’). The genus represents the most inclusive clade containing the type species, *P. judraskeda* ((JX988758; clade P3), but not taxa with the sequence accession numbers HQ868687 (unresolved), EU368015 (clade P1), EU368029 (unresolved), and DQ222877 (clade P2). This is a branch-based definition in which all of the specifiers are extant. The genus *Picomonas* includes at least all taxa in clade P3.

### Type Species. *Picomonas judraskeda* sp. nov

### Picomonas judraskeda sp. nov

#### Diagnosis

Characters of the genus. The oblong cells vary in length between 2.6–3.8 µm, their width is 2–2.5 µm. The longer flagellum measures 12–14 µm; the shorter flagellum 7–9 µm. The longer (anterior) flagellum is oriented towards the anterior; the shorter (posterior) flagellum towards the posterior end of the cell. The nucleus is hemispherical, and over the spherical part of its surface is appressed to the plasma membrane. The feeding apparatus essentially consists of a basket of about 50–60 parallel running fibers (30 nm repeat) of varying lengths that extend from the ventral surface where they are attached to the plasma membrane for up to 1.2 µm towards the dorsal part where they terminate. The basket is open towards the cell’s anterior and dorsal parts, but closed towards the posterior end of the cell. The slit-like cytostome is formed at the ventral cell surface where the ‘side walls’ of the basket fibers connect to the plasma membrane and extends in the anterior-posterior direction for a length of about 1 µm, its width being about 150 nm.

#### Type habitat

Marine plankton.

#### Type locality

Surface water (5 m depth) from Helgoland Roads, (54°11′N, 7°54′E), North Sea, Germany.

#### Type material

The name-bearing hapantotype is a block of resin-embedded cells for electron microscopy (prepared from a single cell-derived culture established from the original natural sample) deposited at the Culture Collection of Algae at the University of Cologne (CCAC; http://www.ccac.uni-koeln.de/), Germany, under the designation PICO.H.001. A parahapantotype block from the same fixation is deposited at the CCAC under the designation PICO.H.002. The culture was subsequently lost and is no longer available.

#### Etymology

Named after its unique stereotypic mode of cell motility, which consists, in succession, of a short fast jump (ju-) into the anterior direction, a slow drag (-dra-) into the opposite direction and an extremely fast and extended movement of the cell away from its original position (skedaddle; -skeda). Although colloquial in the English language and of unknown origin, the name ‘skedaddle’ may be derived from the Greek σκεδασμός (skedasmos,“dispersion”).

## Discussion

### Rationale for the New Protist Phylum Picozoa

The heterotrophic protist *Picomonas judraskeda* gen. nov., sp. nov., described here as a member of a new protist phylum, the Picozoa, has apparently not yet been studied before; the living cell and its morphology by light and electron microscopy were unknown. Gene sequences obtained from environmental clone libraries, however, previously identified a unique pico- or nanoplanktonic eukaryotic lineage that has broad thermal and geographic distribution and became known under the names ‘picobiliphytes’ [34] or ‘biliphytes’ [36]. Originally envisaged as a novel photosynthetic lineage with affinities to katablepharids/cryptophytes [34], [36], recent whole-genome shotgun sequence data of three ‘(pico)biliphyte’ cells sorted by FACS from an environmental sample, did not find any evidence of plastid DNA or of nuclear-encoded plastid-targeted genes in these genomes, and concluded that ‘(pico)biliphytes’ were likely heterotrophic [41]. All previous phylogenetic studies using environmental sequence comparisons, however, agreed that these organisms comprise a genetically unique and diverse novel eukaryotic group to be delineated at a high taxon level. In the recently proposed revised classification of eukaryotes, ‘(pico)biliphytes’ have been placed into “Incertae sedis Eukaryota” [55] and denoted as “Poorly characterized, known only from environmental samples, and no species or genera described.” We established a single cell-derived culture of a ‘(pico)biliphyte’ and characterized it by light and electron microscopy. Our results support the conclusion of Yoon et al. (2011) that these organisms are heterotrophic because no plastids were found. In addition, we revealed a set of highly unusual behavioral and structural features of the cells that to the best of our knowledge have not yet been reported for any other eukaryotic cell. Among these features we list: (1) flagellate cells exhibit a stereotypic pattern of motility consisting of three phases, “jump, drag and skedaddle,” (2) each cell is separated into two parts of almost equal size, an anterior part containing the compartments/organelles typical of a eukaryotic cell, and a posterior part that consists exclusively of vacuoles/vesicles and the feeding apparatus. (3) A single, large vacuolar cisterna physically “seals” both parts of the cell except for a specialized region in which regular projections of the outer mitochondrial envelope, termed “mitovilli,” mediate direct contact between both cell parts. (4) a feeding apparatus consisting of a large basket of fibers that terminate at the ventral cell surface thereby defining the boundaries of a long, slit-like cytostome, which allows formation of a large food vacuole containing only particles of less than 150 nm in size, (5) finally, three of the four microtubular flagellar roots enter the posterior part of the cell, being closely spaced, and terminate near the anterior end of the cytostome.

We feel that these features together with their unresolved position in the eukaryotic phylogenetic tree justify the recognition of this widespread group of marine pico- or nanoplanktonic protists at the phylum level. We recognize, however, that we have investigated only one representative species and don’t know to what extent the structural features described for *Picomonas judraskeda* apply to other members of the phylum. In the diagnosis of the phylum, we therefore make only limited use of the newly discovered structural features, although we believe that most of them will eventually be found to be diagnostic for the phylum when more members have been studied (for the choice of the name Picozoa, see below). The phylogenetic affinities of the Picozoa must remain uncertain at present. We found no evidence for their placement in the novel eukaryotic supergroup Hacrobia [54], which itself has been recently called into question [56] and was also not recovered in our analysis (unpublished observations). Other groups with which the Picozoa have previously been affiliated, namely Telonema, katablepharids/cryptophytes or Plantae [34], [36], [40], [41], [56], in our analyses using the rRNA operon did not show affiliations with the Picozoa, which remained in an unresolved position (unpublished observations). We also conclude that the structural features that characterize *Picomonas judraskeda* bear little resemblance to the cell structures in katablepharids/cryptophytes, Telonema or Plantae [54], [57]–[60].

### Are Picozoa Picoeukaryotes?

Picoplankton was originally defined as those organisms whose cell size lies between 0.2 and 2 µm [5]. Although initially specified as a size fraction ≤ 2 µm, there has never been full agreement about the operational definition of the term picoplankton [61]. Because most field studies, especially those addressing small eukaryotic plankton, have been conducted using 3 µm filters, it has become customary to extend the size range of organisms regarded as picoplankton to those that pass filters of pore size 3 µm, i.e. the size fraction ≤ 3 µm [6], [15]. In lieu of cultures and direct measurements of cell sizes, cell size fractionation through isopore-type membrane filters and epifluorescence microscopy using autofluorescence (in case of photosynthetic cells), often in combination with fluorescence *in-situ* hybridization (FISH) served as a surrogate for cell sizes in ‘uncultured’ organisms. These approaches, however, are not without pitfalls: Initial studies reported that ‘(pico)biliphyte’-specific sequences could be recovered after filtering seawater through either 3 µm [34] or 2 µm [36] filters suggesting that these cells were indeed of ‘pico’-size. Epifluorescence microscopy including FISH and using either whole seawater [36] or cells sorted according to phycoerythrin autofluorescence [34] without pre-filtration through 2–3 µm pore size filters, however, returned signals that depicted irregularly-shaped particles of larger sizes (3.5–6 µm; [34], [36]). These results leave room for interpretation. Fragile cells are known to break during filtration [62], i.e. ‘(pico)biliphyte’ DNA in samples filtered through 2 or 3 µm filters could have originated from larger cells that did not survive the filtration procedure intact. On the other hand, larger fluorescing particles on filters do not necessarily represent only the target cells; they could correspond to cell aggregates or reflect trophic (i.e. phagotrophic) interactions between different partners. That the phycoerythrin-fluorescing particles have misled authors of earlier investigations to conclude that Picozoa were photosynthetic (and hence ‘picobiliphytes’ or ‘biliphytes’) is now obvious (Yoon et al. 2011 and this study), but was already hinted at in a study that reported absence of phycoerythrin fluorescence in North Pacific samples that contained ‘biliphyte’ cells using FISH probes [40].

What do our results contribute to the question of whether Picozoa are picoeukaryotes or not or phrased differently, what can we conclude about the ‘real’ dimensions of these cells? First, we can conclude that not only DNA but live cells of Picozoa can pass through 2 µm filters since we established a culture of *P. judraskeda* from samples pre-filtered in this way. Thus, operationally defined, the Picozoa are picoeukaryotes. However, filter thresholds should not be overinterpreted as flexible cells may pass through pores that are smaller than their actual cell size [63]. Our measurements of the cell sizes of live *P. judraskeda* cells, and of cells fixed and embedded for transmission electron microscopy revealed that cell lengths in interphase cells were quite variable ranging from 2.5–3.8 µm (whereas cell widths were much less variable, see Results). The variability in cell lengths among cells is mainly caused by the variable size of the posterior cell part (PP) that contains the feeding apparatus and the system of ‘digestive’ vacuoles/vesicles and might refer to either different phases of a feeding cycle or a starvation state of the cell (see below). Newly divided cells are also considerably smaller than average cells. It is obvious that the smaller cells of *P. judraskeda* are ≤ 3 µm (2.5×2 µm) and could pass through both 2 or 3 µm filters intact, especially as *P. judraskeda* cells are naked providing additional plasticity to the cells. On average, cell lengths measured in thin-sectioned cells were about 5% less than those measured in live cells, which is to be expected because of minor cell shrinkage during dehydration. The apparently larger cell sizes observed by LM in chemically fixed cells are likely caused by swelling due to osmotic imbalances in the fragile cells when fixed with low glutaraldehyde concentrations. We therefore conclude that cells of *Picomonas judraskeda* fall into the picoplanktonic cell-size range, both operationally by passing through a 2 µm filter, and by direct measurement of cell sizes of live cells. We therefore use the prefix ‘pico’ in the name of the new phylum; although we recognize that the cell dimensions of *P. judraskeda* are at the upper limit of the picoplankton and during feeding transcend the border to the nanoplankton.

### Feeding Behavior and Food Sources

Whereas the heterotrophic nature of the Picozoa is now beyond doubt, their mode of feeding remains essentially unknown. Kim et al. (2011) speculated that phycoerythrin fluorescence in Picozoa may have been the result of phagotrophic feeding of Picozoa on cyanobacteria, e.g. *Synechococcus* spp., whereas Yoon et al. (2011) discussed the possibility that the reported plastid and nucleomorph [34] may have “come from a kleptoplastid or cryptophyte alga captured as food” by Picozoa. In their study on single-cell amplified genomes of three individual cells of Picozoa isolated from seawater Yoon et al. (2011) discovered high abundances of specific single-stranded and double-stranded DNA viruses as well as DNA from marine bacteria of the Bacteriodetes, Proteobacteria, and Firmicutes groups. Furthermore, the three cells differed with respect to the associated viruses and bacteria. The authors concluded that they had studied “complex biotic interactions among previously uncharacterized marine microorganisms,” and regarding one cell, a “virus infection captured *in-situ,*” although they did not rule out passive attachment of viral and bacterial DNA to the surface of the cells. In conclusion, Yoon et al. (2011) suggested that Picozoa might feed on Proteobacteria, Bacteriodetes and large DNA viruses [41].

Do our electron microscope observations shed light on the feeding behavior and the likely food source of the Picozoa? One of the most unusual structural features of *P. judraskeda* is the subdivision of the cell into two parts, an anterior part housing almost all cell constituents and a posterior part, containing what we have identified as a digestive system including the feeding apparatus. Interestingly both parts are separated by a large vacuolar cisterna that leaves larger spaces only for interaction of the posterior digestive body with the single mitochondrion and for passage of three microtubular flagellar roots that presumably position the cytostome (see Results). We serially sectioned 52 cells, but never encountered an intact or recognizable bacterium within the putative food vacuole inside the feeding apparatus, in the posterior digestive body or in any other part of the cell. Because we fixed a growing culture, we would expect to encounter bacteria, if they were a suitable food source for *P. judraskeda*. Although we cannot exclude the possibility that a specific bacterium, that was not present in our bacterized culture, could serve as a selective food item for *P. judraskeda*, there are other reasons to believe that *P. judraskeda* does not, and in fact cannot, feed on bacteria (feeding of *P. judraskeda* on *Synechococcus* spp. can be excluded, because we never encountered chlorophyll/phycoerythrin autofluorescence in sorted cells that were shown to yield ‘(pico)biliphyte’-specific DNA after PCR amplification with the respective primers). We apparently fixed cells for electron microscopy at different stages in the feeding cycle as evidenced by the presence or absence of a food vacuole within the feeding basket (see Results). We measured the width of the cytostome (the region marked by the attachment of the fibers of the feeding basket to the plasma membrane) at these different stages in the feeding cycle and found that it did not change, always being around 150 nm. We conclude that the slit-like cytostome is a rigid structure that cannot take up particles that are larger than its width, thus excluding bacteria. This may also explain the peculiar motile behavior of the cells, which is very unlike that of bacterivorous nanoflagellates [64], [65]. We propose that *P. judraskeda* feeds on particles smaller than 150 nm that are taken up by a fluid phase, bulk flow mechanism. This generates a single food vacuole of enormous size (it can be estimated that the membrane area of a large food vacuole corresponds to about 30–40% of the total plasma membrane surface of the posterior part of the cell) arguing for rapid membrane turnover. At present, we can only speculate about the force(s) that initiate the fluid phase, bulk flow mechanism. The close association of the posterior flagellum with the cytostome slit (see Results) may indicate that flagellar motility (perhaps during the drag movement) could be involved. Regarding the possible food source, we note that the contents are irregular aggregates of ‘fuzzy’ material that resemble <120 nm marine colloids, which are dispersed widely in seawater [66] and may contain lipopolysaccharide material of bacterial membranes [67]. The Picozoa may thus be specially adapted to exploit <120 nm marine colloids as a food source. The ‘skedaddle’ movement could then be envisaged not primarily as a phobic response to escape predators, but rather as a mechanism to explore new food resources once grazing at a specific location has depleted resources. The abundance and spatial distribution of marine colloids may also explain the relatively low number of Picozoa that we observed in our culture of *P. judraskeda* (30–40 cells/ml) as well as the comparable cell numbers reported in natural samples using FISH (55 cells/ml in Not et al. 2007 [34] and up to 300 cells/ml in Cuvelier et al. 2008 [36]). This also suggests that filter-sterilized seawater (0.1 µm filters) could have been the major source of most of the colloidal food particles necessary to support growth of *P. judraskeda*, although a contribution by the bacterial population is also likely. We assume that the unusual structures observed in the mitochondrion (mitovilli and the electron dense inclusions in the intermembrane space) may also be involved in the processing of specific, perhaps mostly lipidic molecules derived from small colloidal food particles. The two prominent microbodies could be involved in the degradation of fatty acids derived from such lipidic molecules.

Could Picozoa perhaps feed on viruses as well as suggested by Yoon et al. (2011)? Viruses constitute the most abundant group of nucleic acid-containing particles in the ocean and up to 10^8^ virus particles per milliliter have been recorded in productive coastal surface waters [68]. Although filtration of natural seawater through 0.1 µm filters would likely exclude the larger size class of marine viruses, the smaller size class (30–60 nm), which is 4- to 10-fold more abundant, would easily pass through a 0.1 µm filter [69]. If we assume that *P. judraskeda* feeds on <150 nm particles by a fluid-phase bulk flow mechanisms, then it is likely that small viruses, such as circular single-stranded DNA viruses (Nanoviridae, Circoviridae; [70]) would be taken up as well. This might explain their prevalence in the single-cell genome amplification of Picozoa [41], although virus particles or DNA attached to the surface of a cell or even co-sorted with such a cell (given the high number of viral particles present in seawater, a sorted droplet of on average 10 picoliters [71], could still contain one or two co-sorted virus particles) should not be dismissed. Although we did not recognize viral particles inside food vacuoles of *P. judraskeda*, we do not exclude the possibility that Picozoa take up small size-class viruses during feeding. Whether these are digested as proposed by Yoon et al. (2011) or exocytosed unaltered during the feeding cycle needs further investigation. We note, however, that the large vacuolar cisterna separating the anterior from the posterior part of the cell would be ideally positioned to prevent access of endocytosed viral particles to the cell’s nucleus.

### Conclusion

During the last decade, culture-independent molecular surveys based on rDNA clone libraries, phylogenetic analyses, and fluorescence *in-situ* hybridization have revealed numerous novel, high-ranking picoeukaryotic (<3 µm) lineages in the oceans. This new knowledge is rapidly altering our understanding of marine microbial food webs, and the biogeochemical significance of marine protists [72]. Although culture-independent techniques have been essential for the discovery of picoeukaryotic biodiversity, for an understanding of the biology of the organisms involved, they should be complemented by studies of the respective organisms in culture. Here, we provided evidence that a genetically diverse and apparently widespread group of picoeukaryotes in the world’s oceans, hitherto known as ‘picobiliphytes’ or ‘biliphytes’, and here formally described as Picozoa phylum nov., displays highly unusual structural and behavioral characteristics that match its isolated position in the eukaryotic phylogenetic tree. Based on the characteristics described for *Picomonas judraskeda* gen. nov., sp. nov., we conclude that Picozoa are heterotrophic and feed on small (<150 nm) particles by a novel fluid-phase, bulk flow uptake mechanism. Further studies on other members of the Picozoa are needed to substantiate this conclusion. We strongly recommend that more effort should be made to cultivate the vast ‘uncultured’ diversity of eukaryotic microbes in the sea.

## Supporting Information

Figure S1 **Electron micrographs of non-consecutive longitudinal serial sections through a** ***Picomonas judraskeda*** **cell from its left to right surface.** A complete reconstruction of the cell shows the absence of a plastid. Numbers in the upper right corners of the respective micrographs correspond to the number of the serial section. A hemispherical nucleus (N) occupied a large volume of the anterior part (AP) of the cell. Approximately, 60% of the surface of the nuclear envelope is closely associated with the plasma membrane. A single mitochondrion (M) with tubular cristae is positioned near the ventral surface of the cell (by definition the surface from which the flagella emerge).The Golgi complex (G) consists of a single Golgi body characteristically located in an anterior groove of the mitochondrion between the nucleus and the flagellar apparatus. Two basal bodies (Ab, Pb) and the flagellar basal apparatus are located near the Golgi body and the mitochondrion at the ventral surface of the cell. Two prominent ‘microbodies’ (MB) are located near the dorsal surface in the AP of the cell. The posterior part of the cell (PP) contains the ‘feeding apparatus’ (cytostome (cy)/feeding basket) and numerous vacuoles/vesicles. A large vacuolar cisterna (vc) separates the anterior from the posterior part (AP/PP) of the cell.
(TIF)

Figure S2 **Unrooted randomized accelerated maximum likelihood (RAxML) phylogenetic tree of partial nuclear SSU rDNA of Picozoa, accessed from Genbank in July 2012.** The sequence (acc no JX988758) from *Picomonas judraskeda* as well as other newly determined sequences (acc no JX988759- JX988767) are highlighted in the tree. Bootstrap values >60% and posterior probabilities >0.80 for the three methods of analyses used (RAxML/NJ by PAUP/MrBayes) are shown on the respective branches. Branches in bold show maximal (100%/1.00) support. Clades marked as ‘P1-P13’, predicted 12 novel picozoan clades supported by bootstrap/posterior probability values (‘clade’ P4 currently remains unresolved). All sequences in the tree are shown with their accession numbers followed by the sample designation as provided in the database.
(EPS)

Table S1 **Primers used for amplification and sequencing of nuclear rRNA operon.**
(TIF)

Movie S1 **Movement of a cell of Picomonas judraskeda.**
(AVI)

Movie S2 **Animation of three dimensional view of Picomonas judraskeda.**
(AVI)

Movie S3 **Animation of three dimensional structure of the feeding apparatus of Picomonas judraskeda.**
(AVI)

## References

[1] Azam F, Fenchel T, Field JG, Gray JS, Meyer-Reil LA, et al (1983) The ecological role of water column microbes in the sea. Mar Ecol Progr Ser 10: 257–263.

[2] Giovannoni SJ, Stingl U (2005) Molecular diversity and ecology of microbial plankton. Nature 437: 343–348. 16163344 10.1038/nature04158

[3] Giovannoni SJ, Vergin KL (2012) Seasonality in ocean microbial communities. Science 335: 671–676. 22323811 10.1126/science.1198078

[4] Strom SL (2008) Microbial ecology of ocean biogeochemistry: a community perspective. Science 320: 1043–1045. 18497289 10.1126/science.1153527

[5] Sieburth JM, Smetacek V, Lenz J (1978) Pelagic ecosystem structure: heterotrophic compartments of the plankton and their relationship to plankton size fractions. Limnol Oceanogr 23: 1256–1263.

[6] Vaulot D, Eikrem W, Viprey M, Moreau H (2008) The diversity of small eukaryotic phytoplankton (≤ 3 µm) in marine ecosystems. FEMS Microbiol Rev 32: 795–820. 18564290 10.1111/j.1574-6976.2008.00121.x

[7] Partensky F, Hess WR, Vaulot D (1999) Prochlorococcus, a marine photosynthetic prokaryote of global significance. Microbiol Mol Biol Rev 63: 106–127. 10066832 10.1128/mmbr.63.1.106-127.1999 PMC98958

[8] Scanlan DJ, Ostrowski M, Mazard S, Dufresne A, Garczarek L, et al (2009) Ecological genomics of marine picocyanobacteria. Microbiol Mol Biol Rev 73: 249–299. 19487728 10.1128/MMBR.00035-08 PMC2698417

[9] Stockner JG, Antia NJ (1986) Algal picoplankton from marine and freshwater ecosystems: A multidisciplinary perspective. Can J Fish Aquat Sci 43: 2472–2503.

[10] Díez B, Pedrós-Alió C, Massana R (2001) Study of genetic diversity of eukaryotic picoplankton in different oceanic regions by small-subunit rRNA gene cloning and sequencing. Appl Environ Microbiol 67: 2932–2941. 11425705 10.1128/AEM.67.7.2932-2941.2001 PMC92964

[11] López-García P, Rodriguez-Valera F, Pedrós-Alío C, Moreira D (2001) Unexpected diversity of small eukaryotes in deep-sea Antarctic plankton. Nature 409: 603–607. 11214316 10.1038/35054537

[12] Moon-van der Staay SY, De Wachter R, Vaulot D (2001) Oceanic 18S rDNA sequences from picoplankton reveal unsuspected eukaryotic diversity. Nature 409: 607–610. 11214317 10.1038/35054541

[13] Jürgens K, Massan R (2008) Protistan grazing on marine bacterioplankton. In Kirchman DL (Ed) Microbial Ecology of the Oceans. Wiley, New York NY, 383–441.

[14] Worden AZ, Not F (2008) Ecology and diversity of picoeukaryotes. In Kirchman DL (Ed) Microbial Ecology of the Oceans. Wiley, New York, NY, 159–196.

[15] Massana R (2011) Eukaryotic picoplankton in surface oceans. Annu Rev Microbiol 65: 91–110. 21639789 10.1146/annurev-micro-090110-102903

[16] Massana R, Balagué V, Guillou L, Pedrós-Alió C (2004) Picoeukaryotic diversity in an oligotrophic coastal site studied by molecular and cultural approaches. FEMS Microbiol Ecol 50: 231–243. 19712363 10.1016/j.femsec.2004.07.001

[17] Sherr EB, Sherr BF (2002) Significance of predation by protists in aquatic microbial food webs. Antonie van Leeuwenhoek 81: 293–308. 12448728 10.1023/a:1020591307260

[18] Chambouvet A, Morin P, Marie D, Guillou L (2008) Control of toxic marine dinoflagellate blooms by serial parasitic killers. Science 322: 1254–1257. 19023082 10.1126/science.1164387

[19] Biegala IC, Not F, Vaulot D, Simon N, Curie M (2003) Quantitative assessment of picoeukaryotes in the natural environment using taxon-specific oligonucleotide probes in association with TSA-FISH (tyramide signal amplification-fluorescent in situ hybridization) and flow cytometry. Appl Environ Microbiol 69: 5519–5529. 12957941 10.1128/AEM.69.9.5519-5529.2003 PMC194996

[20] Not F, Simon N, Biegala IC, Vaulot D (2002) Application of fluorescent in situ hybridization coupled with tyramide signal amplification (FISH-TSA) to assess eukaryotic picoplankton composition. Aquat Microb Ecol 28: 157–166.

[21] Not F, Latasa M, Marie D, Cariou T, Vaulot D, et al (2004) A single species *Micromonas pusilla* (Prasinophyceae) dominates the eukaryotic picoplankton in the western English Channel. Appl Environ Microbiol 70: 4064–4072. 15240284 10.1128/AEM.70.7.4064-4072.2004 PMC444783

[22] Mangot JF, Lepére C, Bouvier C, Debroas D, Domaizon I (2009) Community structure and dynamics of small eukaryotes targeted by new oligonucleotide probes: new insight ubti the lacustrine microbial food web. Appl Environ Microbiol 75: 6373–6381. 19666727 10.1128/AEM.00607-09 PMC2753085

[23] López-García P, Moreira D (2008) Tracking microbial biodiversity through molecular and genomic ecology. Res Microbiol 159: 67–73. 18207371 10.1016/j.resmic.2007.11.019

[24] Cuvelier ML, Allen AE, Monier A, McCrow JP, Messie M, et al (2010) Targeted metagenomics and ecology of globally important uncultured eukaryotic phytoplankton. Proc Natl Acad Sci USA 107: 14679–14684. 20668244 10.1073/pnas.1001665107 PMC2930470

[25] Worden AZ, Janouskovec J, McRose D, Engman A, Welsh RM, et al (2012) Global distribution of a wild alga revealed by targeted metagenomics. Curr Biol 22: R675–R677. 22974991 10.1016/j.cub.2012.07.054

[26] Massana R, Terrado R, Forn I, Lovejoy C, Pedrós-Alió C (2006) Distribution and abundance of uncultured heterotrophic flagellates in the world oceans. Environ Microbiol 8: 1515–1522. 16913912 10.1111/j.1462-2920.2006.01042.x

[27] Kim DY, Countway PD, Gast RJ, Caron DA (2011) Rapid shifts in the structure and composition of a protistan assemblage during bottle incubations affect estimates of total protistan species richness. Microb Ecol 62: 383–398. 21373815 10.1007/s00248-011-9816-9

[28] Weber F, Del Campo J, Wylezich C, Massana R, Jürgens K (2012) Unveilling trophic functions of uncultured protist taxa by incubation experiments in the brackish Baltic Sea. PloS ONE 7 (7): e41970. 10.1371/journal.pone.0041970 PMC3408427 22860041

[29] Sensen CW, Heimann K, Melkonian M (1993) The production of clonal and axenic cultures of microalgae using fluorescence-activated cell sorting (FACS). Eur J Phycol 28: 93–97.

[30] Veldhuis MJW, Kraay GW (2000) Application of flow cytometry in marine phytoplankton research: current applications and future perspectives. Sci Mar 64: 121–134.

[31] Heywood JL, Sieracki ME, Bellows W, Poulton NJ, Stepanauskas R (2011) Capturing diversity of marine heterotrophic protists: one cell at a time. ISME J 5: 674–684. 20962875 10.1038/ismej.2010.155 PMC3105736

[32] Yilmaz S, Singh AK (2012) Single cell genome sequencing. Curr Opin Biotechnol 23: 437–443. 22154471 10.1016/j.copbio.2011.11.018 PMC3318999

[33] Stepanauskas R (2012) Single cell genomics: an individual look at microbes. Curr Opin Microbiol 15: 613–620. 23026140 10.1016/j.mib.2012.09.001

[34] Not F, Valentin K, Romari K, Lovejoy C, Massana R, et al (2007) Picobiliphytes: a marine picoplanktonic algal group with unknown affinities to other eukaryotes. Science 315: 253–255. 17218530 10.1126/science.1136264

[35] Not F, Gausling R, Azam F, Heidelberg JF, Worden AZ (2007) Vertical distribution of picoeukaryotic diversity in the Sargasso Sea. Environ Microbiol 9: 1233–1252. 17472637 10.1111/j.1462-2920.2007.01247.x

[36] Cuvelier ML, Ortiz A, Kim E, Moehlig H, Richardson DE (2008) Widespread distribution of a unique marine protistan lineage. Environ Microbiol 10: 1621–1634. 18341584 10.1111/j.1462-2920.2008.01580.x PMC2408648

[37] Le Gall F, Rigaut-Jalabert F, Marie D, Garczarek L, Viprey M, et al (2008) Picoplankton diversity in the South-East Pacific Ocean from cultures. Biogeosciences 5: 203–214.

[38] Li L, Huang Q, Wu S, Lin D, Chen J, et al (2008) The spatial and temporal distribution of microalgae in the South China Sea: evidence from GIS-based analysis of 18S rDNA sequences. Sci China Ser C: Life sci 51: 1121–1128. 19093087 10.1007/s11427-008-0140-7

[39] Massana R, Pernice M, Bunge JA, Del Campo J (2011) Sequence diversity and novelty of natural assemblages of picoeukaryotes from the Indian Ocean. ISME J 5: 184–195. 20631807 10.1038/ismej.2010.104 PMC3105688

[40] Kim E, Harrison JW, Sudek S, Jones MDM, Wilcox HM, et al (2011) Newly identified and diverse plastid-bearing branch on the eukaryotic tree of life. Proc Natl Acad Sci USA 108: 1496–1500. 21205890 10.1073/pnas.1013337108 PMC3029697

[41] Yoon HS, Price DC, Stepanauskas R, Rajah VD, Sieracki ME, et al (2011) Single-cell genomics reveals organismal interactions in uncultivated marine protists. Science 332: 714–717. 21551060 10.1126/science.1203163

[42] McFadden GI, Melkonian M (1986) Use of Hepes buffer for microalgal culture media and fixation for electron microscopy. Phycologia 25: 551–557.

[43] Geimer S, Melkonian M (2004) The ultrastructure of the *Chlamydomonas reinhardtii* basal apparatus: identification of an early marker of radial asymmetry inherent in the basal body. J Cell Sci 117: 2663–2674. 15138287 10.1242/jcs.01120

[44] Kremer JR, Mastronarde DN, McIntosh JR (1996) Computer visualization of three-dimensional image data using IMOD. J Struct Biol 116: 71–76 http://bio3d.colorado.edu/imod/. 8742726 10.1006/jsbi.1996.0013

[45] Martin-Cereceda M, Roberts EC, Wootton EC, Bonaccorso E, Dyal P, et al (2009) Morphology, ultrastructure, and small subunit rDNA phylogeny of the marine heterotrophic flagellate *Goniomonas* aff. *amphinema* . J Eukaryot Microbiol 57: 159–170. 20015186 10.1111/j.1550-7408.2009.00449.x

[46] Medlin LK, Metfies K, Mehl H, Wiltshire K, Valentin K (2006) Picoeukaryotic plankton diversity at the Helgoland time series site as assessed by three molecular methods. Microb Ecol 52: 53–71. 16703447 10.1007/s00248-005-0062-x

[47] Peer YVD, Wuyts J, Perrie G, Bernard AC (2004) The European ribosomal RNA database. Nucleic Acids Res 32: D101–D103. 14681368 10.1093/nar/gkh065 PMC308799

[48] Zuker M (2003) Mfold web server for nucleic acid folding and hybridization prediction. Nucleic Acids Res 31: 3406–3415. 12824337 10.1093/nar/gkg595 PMC169194

[49] Stamatakis A (2006) RAxML-VI-HPC: maximum likelihood-based phylogenetic analyses with thousands of taxa and mixed models. Bioinformatics 22: 2688–2690. 16928733 10.1093/bioinformatics/btl446

[50] Swofford DL (2002) PAUP*. Phylogenetic Analysis Using Parsimony Version 4. (*and Other Methods). Sinauer Associates, Sunderland.

[51] Posada D, Crandall KA (1998) MODELTEST: testing the model of DNA substitution. Bioinformatics 14: 817–818. 9918953 10.1093/bioinformatics/14.9.817

[52] Ronquist F, Huelsenbeck JP (2003) MrBayes 3: Bayesian phylogenetic inference under mixed models. Bioinformatics 19: 1572–1574. 12912839 10.1093/bioinformatics/btg180

[53] Altekar G, Dwarkadas S, Huelsenbeck JP (2004) Parallel Metropolis coupled Markov chain Monte Carlo for Bayesian phylogenetic inference. Bioinformatics 20: 407–415. 14960467 10.1093/bioinformatics/btg427

[54] Okamoto N, Chantangsi C, Horák A, Leander BS, Keeling PJ (2009) Molecular phylogeny and description of the novel katablepharid *Roombia truncata* gen. et sp. nov., and establishment of the Hacrobia taxon nov. PLoS ONE 4: 11. 10.1371/journal.pone.0007080 PMC2741603 19759916

[55] Adl SM, Simpson AGB, Lane CE, Lukeš J, Bass D, et al (2012) The revised classification of eukaryotes. J Eukaryot Microbiol 59: 429–493. 23020233 10.1111/j.1550-7408.2012.00644.x PMC3483872

[56] Burki F, Okamoto N, Pombert J-F, Keeling PJ (2012) The evolutionary history of haptophytes and cryptophytes: phylogenomic evidence for separate origins. Proc Roy Soc B 279: 2246–2254. 10.1098/rspb.2011.2301 PMC3321700 22298847

[57] Moestrup Ø (2000) The flagellate cytoskeleton: Introduction of a general terminology for microtubular flagellar roots in protists. In Leadbeater BSC, Green JC (eds) The Flagellates. Syst Assoc Special Vol. Ser 59, Taylor & Francis, London, New York, 69–94.

[58] Okamoto N, Inouye I (2005) The katablepharids are a distant sister group of the Cryptophyta: a proposal for Katablepharidophyta disisio nova/Kathablepharida phylum novum based on SSU rDNA and beta-tubulin phylogeny. Protist 156: 163–179. 16171184 10.1016/j.protis.2004.12.003

[59] Shalchian-Tabrizi K, Eikrem W, Klaveness D, Vaulot D, Minge MA, et al (2006) Telonemia, a new protist phylum with affinity to chromist lineages. Proc Roy Soc B 273: 1833–1842. 10.1098/rspb.2006.3515 PMC1634789 16790418

[60] Yabuki A, Inagaki Y, Ishida K (2010) *Palpitomonas bilix* gen. et sp. Nov.: a novel deep-branching heterotroph possibly related to Archaeplastida or Hacrobia. Protist 161: 523–538. 20418156 10.1016/j.protis.2010.03.001

[61] Craig SR (1986) Picoplankton size distributions in marine and fresh waters: Problems with filter fractionation studies. FEMS Microbiol Ecol 38: 171–177.

[62] Worden AZ (2006) Picoeukaryote diversity in coastal waters of the Pacific Ocean. Aquat Microb Ecol 43: 165–175.

[63] Li WKW (1990) Particles in “particle-free” seawater: growth of ultraphytoplankton and implications for dilution experiments. Can J Fish Aquat Sci 47: 1258–1268.

[64] Matz C, Boenigk J, Arndt H, Jürgens K (2002) Role of bacterial phenotypic traits in selective feeding of the heterotrophic nanflagellate *Spumella* sp. Aquat Microb Ecol 27: 137–148.

[65] Erken M, Farrenschon N, Speckmann S, Arndt H, Weitere M (2012) Quantification of individual flagellate-bacteria interactions within semi-natural biofilms. Protist 163: 632–642. 22186014 10.1016/j.protis.2011.10.008

[66] Wells ML, Goldberg ED (1991) Occurrence of small colloids in sea water. Nature 353: 342–344.

[67] Wakeham SG, Pease TK, Benner R (2003) Hydroxy fatty acids in marine dissolved organic matter as indicators of bacterial membrane material. Org Geochem 34: 857–868.

[68] Suttle CA (2005) Viruses in the sea. Nature 437: 356–361. 16163346 10.1038/nature04160

[69] Marie D, Brussaard CPD, Thyrhaug R, Bratbak G, Vaulot D (1999) Enumeration of marine viruses in culture and natural samples by flow cytometry. Appl Environ Microbiol 65: 45–52. 9872758 10.1128/aem.65.1.45-52.1999 PMC90981

[70] Rosario K, Duffy S, Breitbart M (2012) A field guide to eukaryotic circular single-stranded DNA viruses: insights gained from metagenomics. Arch Virol 157: 1851–1871. 22760663 10.1007/s00705-012-1391-y

[71] Sieracki M, Poulton N, Crosbie N (2005) Automated isolation techniques for microalgae. In Andersen RA (Ed) Algal Culturing Techniques. Elsevier, Burlington MA, 101–116.

[72] Massana R, Pedrós-Alió C (2008) Unveiling new microbial eukaryotes in the surface ocean. Curr Opin Microbiol 11: 213–218. 18556239 10.1016/j.mib.2008.04.004

