# Supplementary figures and images for: Picomonas judraskeda Gen. Et Sp. Nov.: The First Identified Member of the Picozoa Phylum Nov., a Widespread Group of Picoeukaryotes, Formerly Known as ‘Picobiliphytes’
Source: PLoS One. 2013 Mar 26;8(3):e59565. doi: 10.1371/journal.pone.0059565 (PMC3608682; doi:10.1371/journal.pone.0059565)

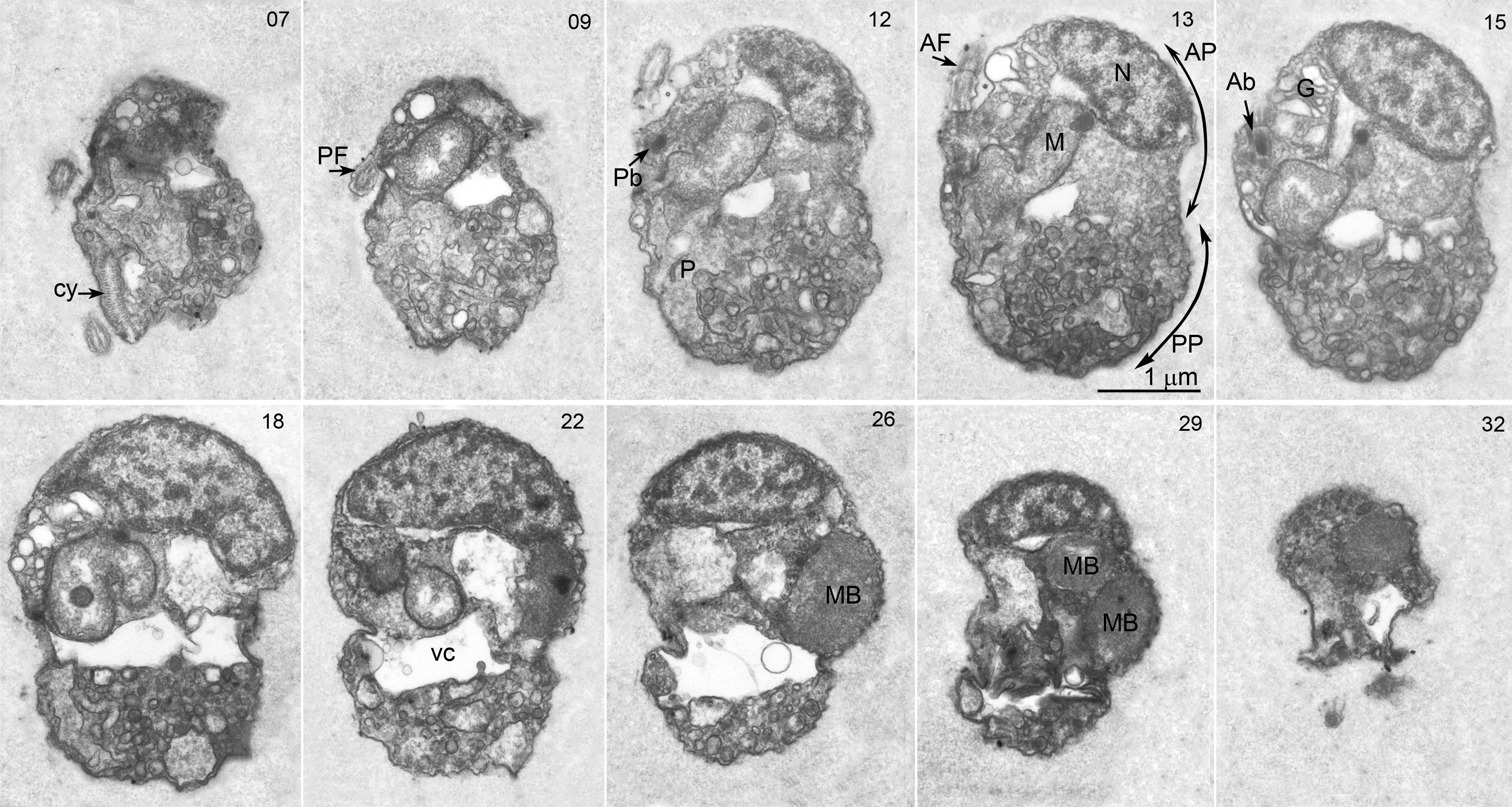

Supplement: Figure S1 — Electron micrographs of non-consecutive longitudinal serial sections through a Picomonas judraskeda cell from its left to right surface. A complete reconstruction of the cell shows the absence of a plastid. Numbers in the upper right corners of the respective micrographs correspond to the number of the serial section. A hemispherical nucleus (N) occupied a large volume of the anterior part (AP) of the cell. Approximately, 60% of the surface of the nuclear envelope is closely associated with the plasma membrane. A single mitochondrion (M) with tubular cristae is positioned near the ventral surface of the cell (by definition the surface from which the flagella emerge).The Golgi complex (G) consists of a single Golgi body characteristically located in an anterior groove of the mitochondrion between the nucleus and the flagellar apparatus. Two basal bodies (Ab, Pb) and the flagellar basal apparatus are located near the Golgi body and the mitochondrion at the ventral surface of the cell. Two prominent ‘microbodies’ (MB) are located near the dorsal surface in the AP of the cell. The posterior part of the cell (PP) contains the ‘feeding apparatus’ (cytostome (cy)/feeding basket) and numerous vacuoles/vesicles. A large vacuolar cisterna (vc) separates the anterior from the posterior part (AP/PP) of the cell. (TIF) [file pone.0059565.s001.tif]

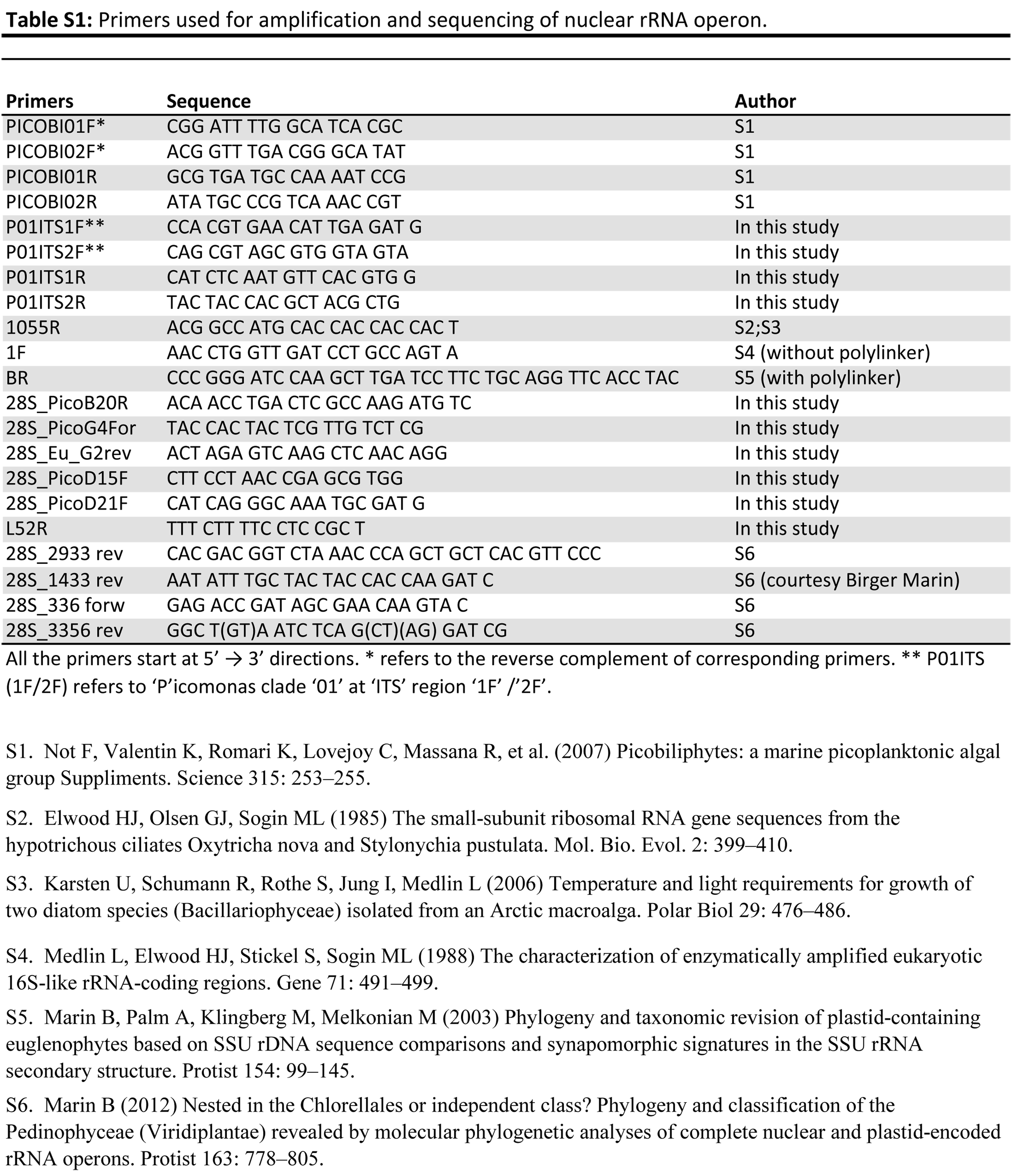

Supplement: Table S1 — Primers used for amplification and sequencing of nuclear rRNA operon. (TIF) [file pone.0059565.s003.tif]
